# The endophytic microbiota of *Citrus limon* is transmitted from seed to shoot highlighting differences of bacterial and fungal community structures

**DOI:** 10.1038/s41598-021-86399-5

**Published:** 2021-03-29

**Authors:** Teresa Faddetta, Loredana Abbate, Pasquale Alibrandi, Walter Arancio, Davide Siino, Francesco Strati, Carlotta De Filippo, Sergio Fatta Del Bosco, Francesco Carimi, Anna Maria Puglia, Massimiliano Cardinale, Giuseppe Gallo, Francesco Mercati

**Affiliations:** 1 Department of Biological, Chemical and Pharmaceutical Sciences and Technologies (STEBICEF), University of Palermo, Palermo, Italy; 2 Institute of Biosciences and Bioresources (IBBR), National Research Council, Palermo, Italy; 3 Department of Life Sciences and Systems Biology, University of Turin, Turin, Italy; 4 Ri.MED Foundation, Palermo, Italy; 5 Laboratory of Mucosal Immunology, Department of Experimental Oncology, European Institute of Oncology, Milano, Italy; 6 Institute of Agricultural Biology and Biotechnology, National Research Council, Pisa, Italy; 7 Institute of Applied Microbiology, Justus-Liebig-University Giessen, Giessen, Germany; 8 Department of Biological and Environmental Sciences and Technologies (DiSTeBA), University of Salento, Lecce, Italy

**Keywords:** Microbiology, Applied microbiology, Microbial communities, Environmental microbiology

## Abstract

*Citrus limon* (L.) Burm. F. is an important evergreen fruit crop whose rhizosphere and phyllosphere microbiota  have been characterized, while seed microbiota is still unknown. Bacterial and fungal endophytes were isolated from *C. limon* surface-sterilized seeds. The isolated fungi—belonging to *Aspergillus*, *Quambalaria* and *Bjerkandera* genera—and bacteria—belonging to *Staphylococcus* genus—were characterized for indoleacetic acid production and phosphate solubilization. Next Generation Sequencing based approaches were then used to characterize the endophytic bacterial and fungal microbiota structures of surface-sterilized *C. limon* seeds and of shoots obtained under aseptic conditions from in vitro growing seedlings regenerated from surface-sterilized seeds. This analysis highlighted that *Cutibacterium* and *Acinetobacter* were the most abundant bacterial genera in both seeds and shoots, while *Cladosporium* and *Debaryomyces* were the most abundant fungal genera in seeds and shoots, respectively. The localization of bacterial endophytes in seed and shoot tissues was revealed by Fluorescence In Situ Hybridization coupled with Confocal Laser Scanning Microscopy revealing vascular bundle colonization. Thus, these results highlighted for the first time the structures of endophytic microbiota of *C. limon* seeds and the transmission to shoots, corroborating the idea of a vertical transmission of plant microbiota and suggesting its crucial role in seed germination and plant development.

## Introduction

Recent evidences allowed considering the plants as a single unit of evolution, called “holobiont” that includes plant and its associated microbiota^1^. In addition, the plants showed a high heterogeneity for morphological and physiological traits and, therefore, they can be also considered as a mosaic of microhabitats. Some of these microhabitats were well characterised—such as rhizosphere^2^, phyllosphere^3^, as well as shoot- and root-endosphere^4^—but other remained relatively unknown, and were much less studied, mostly due to the low bacteria abundance compared to other tissues.

Seed, the organ of generational and genetic renewal and recombination in plants, belongs to less-known habitat category, although it is known to be a source of endophytic bacteria both in wild and domesticated plants^5–7^. Seed microbiota members colonize the surface (epiphytic microbiota) or reside in internal seed tissues (endophytic microbiota)^7^. Historically, the presence of microorganisms in seeds has been relegated to only transient association of little relevance to the plant. Current evidences suggest that the microbiome that take place in, on and around seeds can have direct impacts on seed quality. Bacterial endophyte members usually belong to *Proteobacteria*, *Firmicutes*, *Actinobacteria* and *Bacteroidetes* phyla and their roles are still unclear. Interestingly, different seed-borne endophytes are able to produce a plethora of plant growth-promoting (PGP) factors—such as indoleacetic acid (IAA), 1-aminocyclopropane-1-carboxylate deaminase, and acetoin—and carry out metabolic activities useful to plants such as nitrogen fixation and phosphate solubilization^6,8^. In addition, a number of diverse seed endophytes have proven to inhibit different phytopathogenic fungi by producing antifungal compounds, toxins, or hydrolytic enzymes, thereby strongly influencing the first steps of the plant’s life^9^. These evidences sustain the hypothesis of a dynamic plant-bacterial co-evolution and functional selection^1,7,8^.

The knowledge of the endophytic communities residing in seeds has grown tremendously in recent years^5,8, 10,11^. Most studies, however, have focused on the composition and structure of microbiota in short-lived annual herbaceous plants, while few investigations have focused on the question regarding the structure and assembly of seed microbiome in woody perennial plants^5^. Microorganisms from surface sterilized seed were also reported in different commercial crops such as coffee, grapevine, bean, rice, maize, wheat and tomato^11^; however Newcombe et al.^8^ underlined only one or none cultivable bacterial or fungal endophyte in surface-sterilized seeds of 98 plant species, belonging to 39 families. Metabarcoding approaches showed endophyte high diversity in bulked seed samples^12^ rather than in individual seeds, suggesting a putative limit in plant microbiome diversity, due to both the host resistance and inhibitory interactions among seed endophytes^13^. Seed bacterial endophytes quickly colonize other tissues (roots and shoots), underlining their ability to colonise, survive and to be transmitted to the next generation plants^6^. This vertical transmission, defined as the direct transfer from two different generations, should promote the selection of beneficial endosymbionts against pathogens and adverse environmental conditions, supporting the hosts during germination and seedling development^11^.

Many reports highlighted the presence of fungal endophytes in seeds, while only few studies described the presence and role of bacterial seed endophytes during germination and seedling development^10,11^. Despite the beneficial properties on plant development and health^11^, supporting the host’s fitness^14^, and the relative potential applications, the composition of seed endophytic communities and their role are still largely unexplored in many species and the comprehension about plant–microbe interaction is compounded by its complexity^6^.

Except the work by Newcombe et al.^8^, an exploratory summary reporting the presence of one cultivable endophyte *per* seed in *Rutaceae* plant family, no studies have explored the citrus crop seed microbiota. Citrus is the most economically important evergreen subtropical fruit crop in the world, producing nearly 125 million tons of fruits with more than 9 million hectares of cultivated fields^15^. All citrus fruits are an important source of bio-compounds contributing to supply the human nutrition. In citrus, the seed-to-seedling phase is very crucial. The seedling is commonly used as rootstock onto which scion varieties are grafted; it faces limitations and deficiencies of habitats, enhances the tolerance against abiotic stresses, provides tolerance to pests and diseases and strongly influences the plant health and productivity^16^.

In this work, we investigated and characterized for the first time the seed endophytic microbiota of *Citrus limon* (L.) Burm F. (“Femminello” lemon) by culture-dependent and Next Generation Sequencing (NGS)-based approaches. In particular, the isolated endophytic bacterial and fungal strains were characterized on the base of their 16S rRNA gene and rRNA gene ITS region sequences, respectively, and for the capability of producing IAA and phosphate solubilization. In addition, the structure of seed endophytic bacterial and fungal community was characterized by NGS analysis of 16S rRNA gene and rRNA gene ITS region libraries, respectively, and was also confirmed by Fluorescence In Situ Hybridization (FISH) coupled with Confocal Laser Scanning Microscopy (CLSM). Noteworthy, by NGS-based approach and FISH-CLSM analysis we highlighted the transmission of seed endophytes to shoots obtained from germinated surface-sterilized seeds, outlining the way for future host/endophytes relationship studies during plant development.

## Methods

### Plant material and sampling procedure

Fruits of *Citrus limon* L. Burm were collected at the IBBR-CNR experimental field located in the Collesano district (Palermo), Italy (37°59′ 19.9″ N, 13°54′ 55.8″ E, 80 m a.s.l.). In total, 30 fruits were collected randomly from three different mother plants (i.e. 10 fruits from each tree). Seeds were extracted under aseptic conditions at fruit maturity, dried and stored at 4 °C until use. Shoots were excised under aseptic conditions from seedlings growing in vitro and regenerated from surface-sterilized seeds.

### Isolation and identification of endophytic microorganisms from *C. limon* seeds

A total of 9 seeds, obtained as above described, were randomly chosen and divided into 3 falcon tubes containing 3 seeds from each mother plant (Supplementary Fig. S1). These seeds were surface-sterilized following the procedures described by Alibrandi et al.^5^ with minor modifications. In particular, immersion in sterile distilled water for 5 min was followed by subsequent immersion into 70% (v/v) ethanol (1 min), 2.5% (w/v) sodium hypochlorite solution (2 min) and, finally, into 70% (v/v) ethanol (1 min). Afterwards, the seeds were repeatedly rinsed using sterile distilled water. To confirm that the sterilization process was successful, 1 mL aliquots of the last-step washing water were plated on different growth agar-media (*i.e.* Luria Bertami (LB), Mannitol Soya flour (MSF)^17^ and R2YED^17^) and examined for microbial growth after incubation at 30 °C for 7–15 days using aliquots of washing water of unsterilized *C. limon* seeds as control (Supplementary Fig. S1).

The surface-sterilized seeds, still divided into 3 groups of 3 members from each mother plant, were immersed into sterile distilled water for 1 h. Then, to obtain a homogenate containing endophytes, each group of seeds was grounded using a Potter–Elvehjem Tissue Grinder (Sigma-Aldrich, St Louis, USA). The homogenate was resuspended in 2 mL phosphate saline buffer (PBS: 140 mM NaCl, 3 mM KCl, 10 mM Na_2_HPO_4_, 2 mM KH_2_PO_4_ pH 7.4) and finally shaken at 150 r.p.m. for 1 h. Several aliquots (100 µL) were then plated on different culture LB, MSF, R2YED agar-media. The plates were incubated at 30 °C for 7 days (Supplementary Fig. S1). The bacterial and fungal colonies obtained were selected on the basis of colony morphology and/or pigmentation and repeatedly incubated on agar-media to obtain pure cultures. The isolated bacteria were taxonomically characterized on the basis of their 16S rRNA gene sequence, while identification of isolated fungi was based on rRNA gene ITS1-5.8S-ITS2 (ITS1-2) region sequence, using the universal bacterial primers 27F and 1492R^18^ and the fungal primers ITS1F and ITS4^19^, respectively, by colony PCR assay as previously described^20^. The PCR products were purified by using a GenElute Plasmid MiniPrep (Sigma-Aldrich, St Louis, USA) and sequencing was performed by BMR genomics (www.bmr-genomics.it). The bacterial and fungal amplicon sequences—reconstructed from raw forward and reverse sequence data by FinchTV software (Perkin Elmer) and analysed using the DECIPHER Find Chimeras web tool (http://decipher.cee.wisc.edu/FindChimeras.html)—were submitted to GenBank with the following accession numbers: MN968814, MN968815, MN968816, MN960410, MN960411, MN960412, MN960413. The reconstructed sequences were used for a NCBI nBLAST interrogation against (i) “16S ribosomal RNA sequences” (type material only) for bacterial sequences or against (ii) “Internal transcribed spacer (ITS) from Fungi type and reference material” for fungal sequences. The first ten hits for each interrogation were selected and the FASTA files were used to perform a phylogenetic analysis by R/adegenet package^21^, using the Neighbor-Joining (NJ) and Nei’s distances^22^. Confidence for tree topologies was estimated by bootstrap values based on 1000 replicates and the dendrogram was plotted by using R/ggtree package^23^. For phylogenetic analysis of bacterial and fungal strains, the 16S rRNA gene sequence of *Escherichia coli* strain NBRC 102203 and the ITS1-2  region sequence of *Candida maltosa* CBS 5611 were used as outgroups, respectively. In addition, the reconstructed 16S rRNA gene and ITS1-2 sequences were also analyzed by SILVA “Alignment, Classification and Tree” (ACT) Service^24^ and by UNITE nBLAST^25^ tools, respectively.

### IAA production

To test IAA production by isolated endophytes, a colorimetric assay was performed using the Salkowski’s reagent (0.5 M FeCl_3_ in 35% HClO_4_ aqueous solution)^26^. In particular, the strains were grown in 3 mL of LB medium and LB medium supplemented with tryptophan (0.1 mg/mL), and incubated for 1 and 6 days (28 °C, 200 rpm). The cultures were centrifuged after incubation and supernatants were mixed with Salkowski’s reagent (1:2). The optical density (OD) was recorded at 530 nm after 30 min of incubation in the dark using IAA (Sigma-Aldrich) solutions as standard.

### Inorganic and organic phosphate solubilization

In order to characterize the ability to solubilize organic and inorganic phosphates, all isolated endophytes were plated on NBRIP agar media^27^ containing different sources of phosphate: AlPO_4_ (5 g L^−1^), Ca_3_P_2_O_8_ (5 g L^−1^); FePO_4_ (5 g L^−1^); phytate (2 g L^−1^). In particular, the strains were pre-incubated using 3 mL TBS medium (200 rpm, 30 °C). Then, 50 µL of microbial suspension (1 OD_600_) were plated on NBRIP agar media to check the development of a solubilisation halo around the colonies. To highlight phosphate solubilization by clearance halo formation, the growth media were added with bromophenol blue (0.05 g L^−1^) but Ca_3_P_2_O_8_ containing one.

### Total DNA extraction and NGS-based analysis

A total number of 30 *C. limon* seeds (10 from each of the three mother plants) were separated into two aliquots of 15 seeds each and surface-sterilized using the procedures above described but step 4 (Supplementary Fig. S1). The two aliquots of surface-sterilized seeds were immersed separately into liquid nitrogen and ground using a Potter–Elvehjem Tissue Grinder in sterile condition. Then, 100 mg of each powdered aliquot were used to extract metagenomic DNA (Supplementary Fig. S2), using the Genomic DNA purification kit (Thermo Scientific, Waltham, USA), following the manufacturer’s instructions.

*C. limon* shoots were obtained (approximately 15 days after germination) from surface-sterilized seeds incubated in vitro on Murashige and Skoog (MS) solidified (7 g L^−1^ agar) medium^28^ supplemented with 50 g L^−1^ sucrose as carbon source. Seeds were maintained in a climate chamber at 25 ± 1 °C with 16 h photoperiod and photosynthetic photon flux of 100 µmoL m^−2^ s^−1^. Then, 30 shoots (obtained from 10 surface-sterilized seeds collected from each of the three mother plants) having a size of approximately 10 cm in length, were excised, separated into two aliquots of 15 shoots, and homogenized in liquid nitrogen as above described. Then, 100 mg of each powdered aliquot were processed in order to extract metagenomic DNA by following the same procedures described above for surface-sterilized seeds (Supplementary Fig. S2). Extracted DNAs were subjected to a quality test performed by PCR to amplify the 16S rRNA gene as described above for the identification of endophytic bacteria.

NGS was performed by IGA Technology Services (Udine, Italy) using MiSeq (Illumina, San Diego, CA, USA) to generate 300 bp paired-end reads. In particular, PNA clamps and 515f/806r primer pair were used to amplify 16S rRNA geneV4 region^29^ and ITS3/ITS4 primer pair to amplify DNA region containing ITS2^30^.

Taxonomical classification based on 16S rRNA gene V4 and fungal ITS2 sequences were carried out using the Mothur package (Galaxy Version 1.39.5.0) within a Galaxy environment taking advantage of the European server (https://usegalaxy.eu/), following the standard operating procedure^31^. Sequences were qualitatively checked and trimmed by the means of Trimmomatic (Galaxy Version 0.36.0)^32^. Then, forward and reverse reads were combined using the MAKE.CONTIGS command (Galaxy Version 1.39.5.0). Reads that contained ambiguous bases or had long homopolymer stretch (> 7 bases) were excluded from analysis. The reads derived from 16S rRNA gene were aligned against the SILVA v.132 reference database^24^ and misaligned sequences were considered of poor quality and deleted. Preliminary clustering was performed by the PRE.CLUSTER command (Galaxy Version 1.39.5.0), and sequences showing different ≤ 2 nucleotides were merged. Chimeras were removed using CHIMERA.VSEARCH (Galaxy Version 1.39.5.1). Resultant 16S rRNA gene sequences were classified by CLASSIFY.SEQ command (Galaxy Version 1.39.5.0) using SILVA seed v.132 reference database^24^ and taxonomy files, removing sequences than showed homology to chloroplast and mitochondrial plant genomes. Fungal ITS2 sequences were classified also by CLASSIFY.SEQ command (Galaxy Version 1.39.5.0) using the UNITE database^25^.

Cluster analysis was carried out using Galaxy (Version 1.39.5.0). Sequences were clustered into OTUs using the CLUSTER.SPLIT (taxonomic level 4 with a cut-off of 0.03) and classified through CLASSIFY.OTU command. Fungal singletons have been removed by the REMOVE.RARE command. Rarefaction and alpha diversity were assessed by RAREFACTION.SINGLE and SUMMARY.SIGLE commands and beta diversity was calculated by DIST.SHARED.

To perform an analytic comparison between seed- and shoot-associated microbiota, alpha-diversity indices were calculated by the SUMMARY.SIGLE command (Galaxy Version 1.39.5.0)—namely species observed (SOB, *i.e.* the number of observed species), Chao 1 richness (the hypothetical total number of OTU), Shannon diversity and the Simpson diversity (reported as the inverse of the classical Simpson diversity estimator-invsimpson). Beta-diversity of bacterial and fungal community structures was calculated using the Jaccard similarity coefficient—based on the observed richness (jclass)—and the Yue and Clayton theta similarity coefficient (thetayc) by DIST.SHARED command (Galaxy Version 1.39.5.0). The PERMANOVA statistical test was calculated between seeds and shoots, either for bacterial or fungal microbiota structures, using JCLASS and thetaYC distances by the QIIME_compare_categories version 1.9.1.0 in https://usegalaxy.eu with 999 permutations for calculating statistical significance.

The microbial community has been visualized using Krona^33^ and Phinch^34^. NGS data are available at the European Nucleotide Archive (https://www.ebi.ac.uk/ena) with the accession identifier PRJEB34309.

### Fluorescence In Situ Hybridization-Confocal Laser Scanning Microscopy (FISH-CLSM)

Surface-sterilized seeds and stem sections of shoots regenerated in vitro from surface-sterilized seeds were embedded in tissue freezing medium Jung (Leica Instruments GmbH, Nussloch, Germany). Cryosections of 30 µm were obtained using the low-temperature constant-cooling cryostat HM 500 OM (MICROM, Walldorf, Germany) at − 20 °C; the cryosections were gently washed in phosphate-buffered saline 1x (PBS) to remove the embedding medium and fixed in 3:1(vol/vol) 4% paraformaldehyde in PBS for 6 h at 4 °C, then washed three times in ice-cold PBS (for 10/20/30 min stepwise, at 4 °C), and finally stored at − 20 °C in 1:1 (v:v) ice-cold PBS:ice-cold 96% ethanol until FISH staining.

To reveal bacterial endophytes, the cryosections were stained by in tube-FISH according to Cardinale et al.^35^, using the Rhodamine- or Cy3-labeled EUB338MIX probe (the universal probes for bacteria made of an equimolar mixture of EUB338, EUB338II and EUB338III probes^36,37^) in combination with the following specific probes: the Cy5-labelled HGC236 probe, specific for *Actinobacteria*^38^; the FITC- or Cy5-labelled LGC354MIX probe, specific for *Firmicutes* (made of an equimolar mixture of LGC354A, LGC354B and LGC354C^39^); the Cy5-labelled GAM42a probe, specific for *Gammaproteobacteria*^40^; and the ATTO488-labelled BET42a probe, specific for *Betaproteobacteria*^40^. Hybridization was performed at 42 °C for two hours in the dark, followed by washing at 43 °C. Stained samples were dipped for 5 s into ice-cold water, placed on a glass slide, dried out with soft compressed air, immediately mounted with antifade reagent, covered with a coverslip and finally sealed with nail polish. The probe combinations used were: EUB338MIX + LGC354MIX + HGC236 and EUB338MIX + Gam42a + Bet42a, labelled with fluorochromes with non-overlapping spectra. The occurrence of false positive signals derived from aspecific adhesion of FISH probes or fluorochromes to seed or shoot tissues was checked by staining a subsample with a mixture of Cy3-, Cy5-, and FITC-labelled NONEUB probes^41^.

Stained samples were observed with the confocal laser scanning system Leica SP8 (Leica Microsystems GmbH, Mannheim, Germany). Four confocal channels were acquired, one for each of the Cy3-, Cy5- and FITC-conferred signal, plus one further channel for the autofluorescence of the seed tissues (excitation: 405 nm, emission: 420–480 nm). Confocal stacks were acquired with a Leica 63X 1.0 NA water-immersion objective, by applying a Z-step of 0.6–0.8 µm. Maximum projections, volume-renderings and three-dimensional models were created with the software Imaris version 8.3 (Bitplane, Zurich, Switzerland). Final figures were assembled with Adobe Photoshop CS6 (Adobe Systems Inc., San Jose, USA).

## Results

### Isolation and characterization of *C. limon* seed microbial endophytes

**Figure 1:**
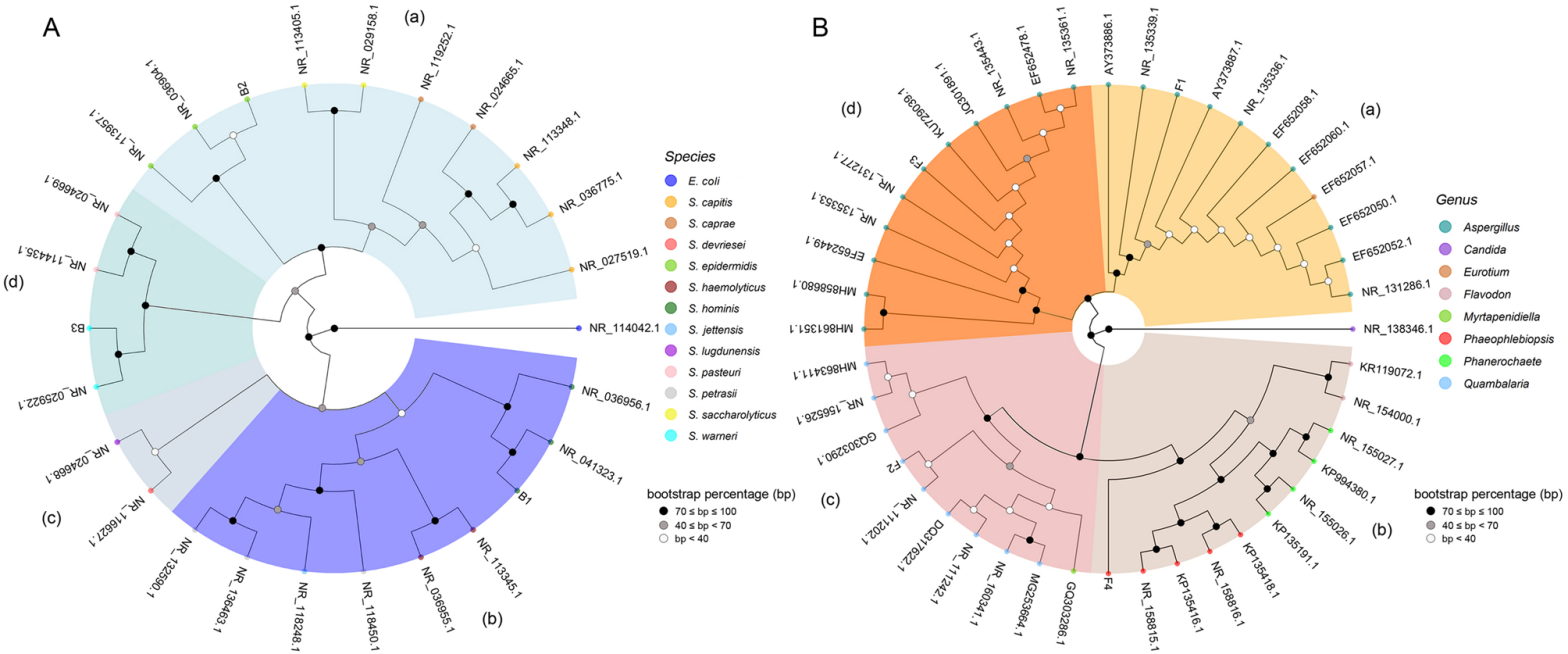
Bacterial (**A**) and fungal (**B**) phylogenetic tree based on sequences of 16S rRNA gene and of ITS1-2 region, respectively, derived from *C. limon* seed endophytes and sequences retrieved from the NCBI database according to BLAST interrogation for sequence homology. (**A**) B1–3: bacterial endophytes isolated from *C. limon* seeds. (**B**) F1–4: fungal strains isolated from *C. limon* seeds. The branching pattern was generated by NJ method and Nei’s distance. The species (**A**) and genera (**B**) analyzed were highlighted using colors reported in legends. Different clusters (a, b, c and d) and bootstrap values (1000 replicates) were indicated in the figure. The sequences of the *Escherichia coli* 16S rRNA gene (**A**) and *Candida maltose* ITS1-2 region (**B**) were used as outgroups, respectively.

Based on serial dilution and colony counting, the concentration of microbial endophytes ranged between 10^3^–10^4^ CFUs/g. A total of 60 endophyte colonies were randomly picked and re-streaked on fresh agar plates. The isolated endophytes were preliminarily grouped by using phenotypic criteria. The isolates with distinctive colony morphology and/or pigmentation were selected for full-length sequencing of the 16S rRNA gene or ITS1-2 containing DNA region, allowing to identify three bacterial and four fungal strains, respectively. The NJ cluster analysis, based on the pair-wise distance matrix among bacterial strains, was able to identify four main groups (Fig. 1A). All bacteria—referred as B1–3, respectively—belonged to the genus *Staphylococcus* (Fig. 1A). The phylogenetic analysis demonstrated evolutionary correlations of the isolated endophytes with *S. hominis* (B1 strain), *S. epidermidis* (B2 strain) and *S. warneri* (B3 strain) (Fig. 1A)*.* The correlation with the *Staphylococcus* genus for B1-3 strain was also confirmed by ACT service analysis (Supplementary Table S1). Phylogenetic analysis was also performed using the sequences of fungal strains- named as F1–4, respectively. This analysis allowed to correlate the F1 and F3 strains with *Aspergillus* and F2 strain with the *Quambalaria* genera, respectively (Fig. 1B). On the contrary, the F4 strain did not correlate univocally with one genus by NJ cluster analysis based on NCBI nBLAST retrieved sequences (Fig. 1B). Indeed, UNITE nBLAST analysis confirmed the above described taxonomic correlation for F1-3 strains and allows to correlate the F4 strain with the *Bjerkandera* genus (Supplementary Table S2).

In addition, the isolated endophytes were characterized for the capability of solubilizing phosphate and producing IAA. The F2 and F4 isolates were no more able to grow after storing at − 20 °C in glycerol solution. Anyhow, the assays revealed that all the tested strains were able to grow on solid media containing different sources of inorganic (i.e. Ca-, Fe- and Al-) or organic (i.e. phytate) phosphate. However, no clear and neat solubilization halo was observed around microbial colonies grown on Ca- and Al-phosphate containing media. On the contrary, clear solubilization halos, highlighted by a colour change from blue to yellow, were observed around B1 and F3 isolate colonies grown on phytate and Al-phosphate, respectively (Table 1), revealing the capability of producing diffusible phosphate-solubilizing compounds.

**Table 1:** IAA production and phosphate solubilization by microbial endophytes isolated from *C. limon* seeds.

Strain	IAA production^a^	Phosphate solubilization^b^			
		Alluminium	Calcium	Iron	Phytate
B3	–	g	g	g	g
B2	2.1 ± 0.1	g	g	g	g
B1	4.8 ± 0.2	g	g	g	g+
F1	–	g	g	g	g
F2	6.7 ± 0.1	g	g	g+	g

^a^Reported as µg/µL of IAA equivalents, − no production.

^b^*g* growth, + clearance halo.

### Microbial endophytic community structure of *C. limon* seeds and shoots

**Figure 2:**
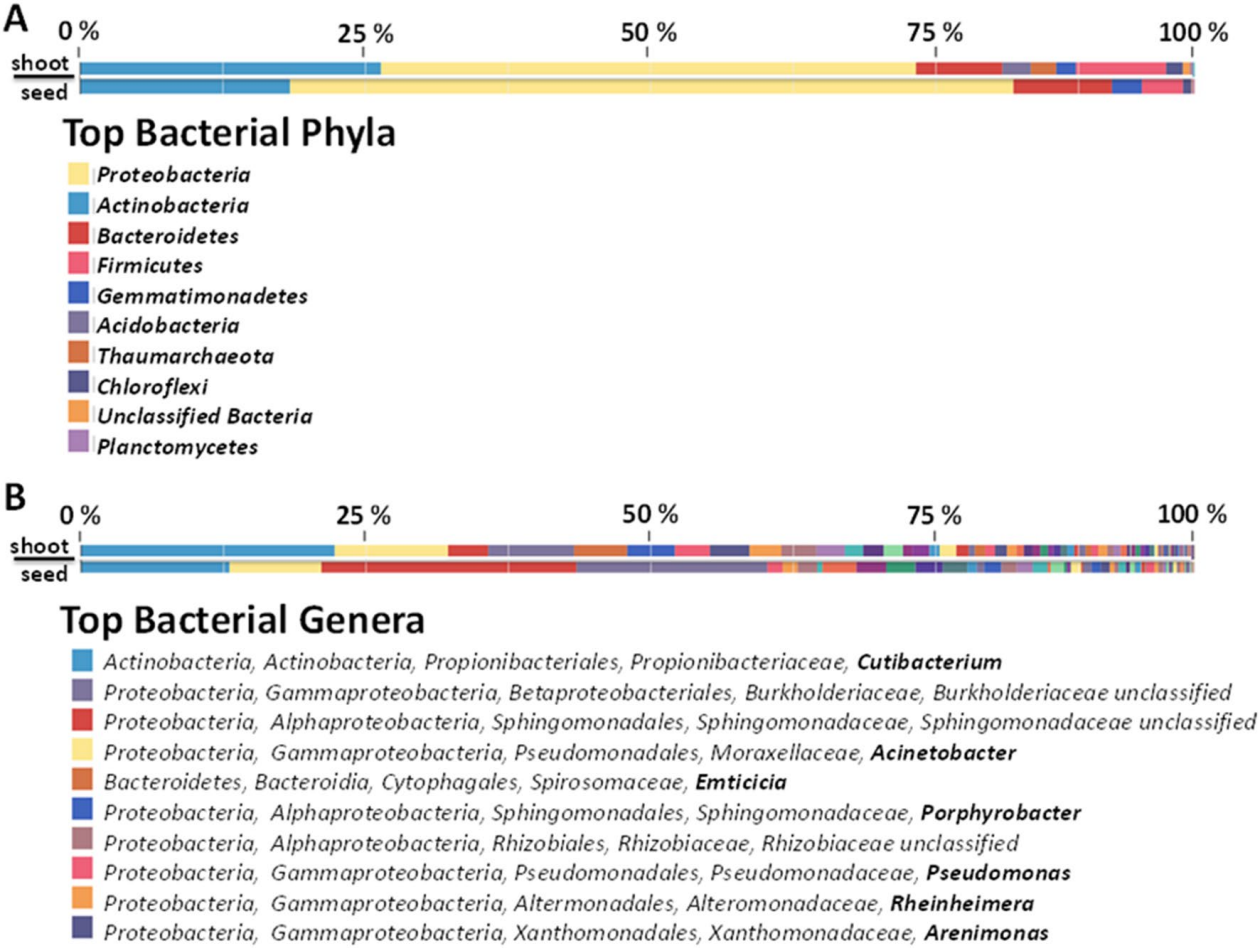
Relative quantification of bacterial phyla (**A**) and genera (**B**) of *C. limon* seed and shoot microbiota, as detected by NGS sequencing. In panel B, bacterial genera are highlighted in bold.

**Figure 3:**
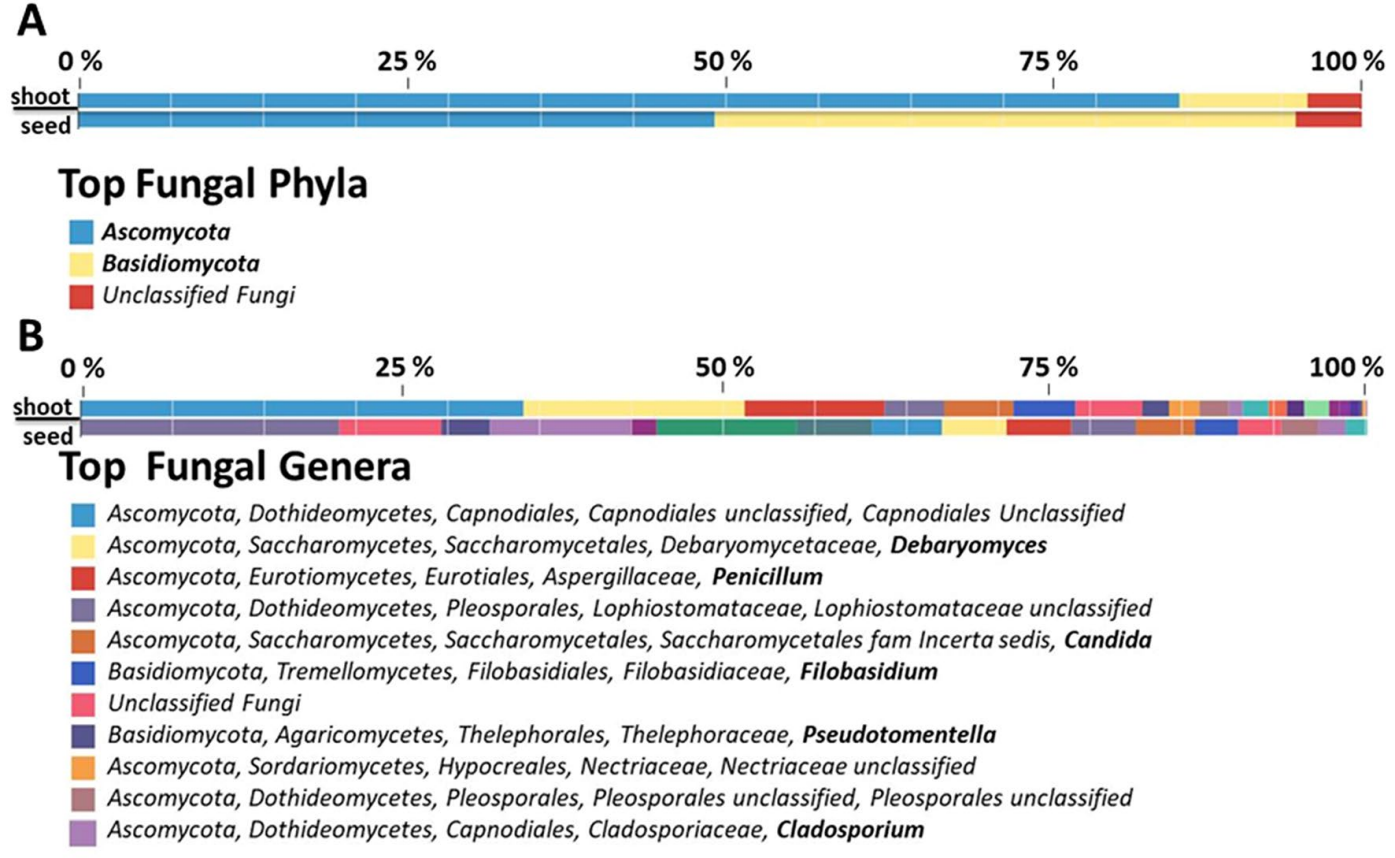
Relative quantification of fungal phyla (**A**) and genera (**B**) of *C. limon* seed and shoot microbiota, as detected by NGS sequencing. In panel B, fungal genera are highlighted in bold.

To obtain a more comprehensive picture of microbial endophyte structure of *C. limon* seed, NGS-based analyses were performed using the metagenomic DNA extracted from surface-sterilized seeds. In particular, NGS was carried out on 16S rRNA gene resulting in 156,606 raw reads, nearly 74% of which (115,785) passed the quality filtering. After the removal of mitochondrial and chloroplast sequences, 2,386 reads were classified as derived from bacteria (Supplementary Table S3). Sequence homology analysis highlighted that the most represented bacterial phyla were *Proteobacteria* (65%), *Actinobacteria* (19%), *Bacteroidetes* (9%) and *Firmicutes* (4%) (Fig. 2A) as well as the most abundant bacterial genera were *Cutibacerium* (13%) and *Acinetobacter* (8%) (Fig. 2B). On the other hand, the NGS analysis of fungal ITS region resulted in 344,189 reads, and nearly 91% of them (314,485) passed the quality filtering. Almost 99% of reads showed homology to plant ITSs, and only 232 sequences were classified as fungi (Supplementary Table S4). However, sequence homology allowed to group them in two main phyla, *i.e. Ascomycota* (50%) and *Basidiomycota* (45%) (Fig. 3A), while the most representative genus was *Cladosporium* (11%) (Fig. 3B).

To outline the fate of *C. limon* seed microbial endophytes, post-germination tissues were also characterized by NGS-based analysis. Stem of shoots, obtained from surface-sterilized seeds growing in sterile conditions, were processed to obtain total DNA that was used to perform a NGS analysis of bacterial 16S rRNA genes and of fungal ITS region, as above described. From bacterial 16S rRNA gene sequencing 181,537 filtered reads (nearly 87%) out of 239,654 raw reads were obtained. After mitochondrial and chloroplast 16S rRNA gene plant sequence removal, 3907 were classified as bacteria (Supplementary Table S5). In agreement to seed endophyte community structure, the most represented bacterial phyla were *Proteobacteria* (48%), *Actinobacteria* (27%), *Firmicutes* (8%) and *Bacteroidetes* (8%), (Fig. 2A) as well as *Cutibacterium* (23%), and *Acinetobacter* (10%) were the most abundant bacterial genera (Fig. 2B). Similarly, 527,761 filtered reads (out of 532,174 raw reads) were obtained by ITS region sequencing. After plant ITS read removal, 2,775 were classified as derived from fungi (Supplementary Table S6). The most represented fungal phyla were *Ascomycota* (86%) and *Basidiomycota* (10%), in agreement to the result obtained from seeds (Fig. 3). Furthermore, except an unclassified genus belonging to *Capnodiales* family, the most abundant fungal genera were *Debaryomyces* (17%) and *Penicillium* (11%) (Fig. 3B).

Interestingly, the seed- and shoot-associated microbiota comparison, performed by using both Shannon and Simpson diversity indexes, highlighted an increasing of diversity in the shoots for bacteria community while an opposite trend has been observed for fungal components (Supplementary Table S7). Anyhow, beta-diversity index underlined a general similarity (jclass > 0.8; thetayc > 0.7) between seed and shoot communities for both bacteria and fungi (Supplementary Table S8). This aspect was further confirmed by PERMANOVA (Supplementary Table S9). Indeed, 48% and 46% of bacterial and fungal endophyte reads—consisting in the 17% and 14% of bacterial and fungal OTUs, respectively—are shared between the two tissues analyzed (Supplementary Fig. S3), and this implies that the most represented endophytes are in common between seeds and shoot tissues, while the major differences are due to rare OTUs.

### Localization of *C. limon* bacterial endophytes in seeds and shoot tissues

**Figure 4:**
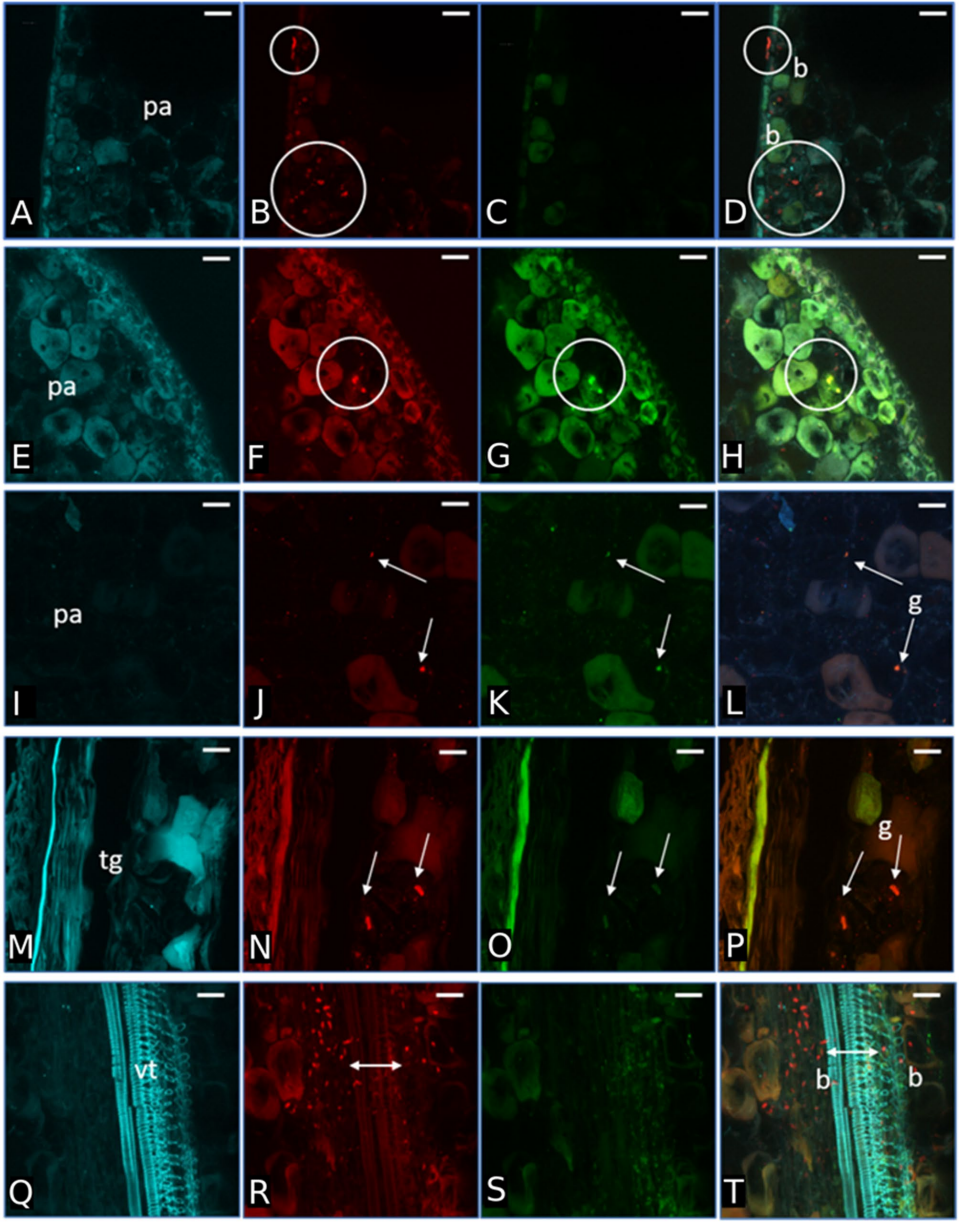
Bacterial colonization of *C. limon* seeds. Confocal laser scanning microscopy images showing Fluorescence In Situ Hybridization (FISH)-stained endophytic bacteria of *Citrus limon* seed cryosections. First and second row show the result after staining with the Rhodamine red-labelled universal FISH-probes EUB338MIX (shown as red in panel (**B**) and (**F**), circles) and the Cy5-labelled Firmicutes-specific probe LGC354MIX (shown as green in panel G, circles); panel (**D**) and (**H**) are the overlaps of the images (**B**, **C**) (cells stained by the universal probe EUB338MIX only) and (**F**–**G**), respectively (circle in the panel H indicates *Firmicutes* cells); third, fourth and fifth row show the result after staining with the Rhodamine red-labelled universal FISH-probes EUB338MIX (shown as red in panel (**J**), (**N**) and (**R**), arrows) and the Cy5-labelled *Gammaproteobacteria*–specific probe GAM42a (shown as green in panel **K** and **O**, arrows); panel (**L**), (**P**) and (**T**) are the overlap of the images (**I**–**K**), (**M**–**O**) and (**Q**–**S**) respectively (arrows in the panels (**L**) and (**P**) indicate *Gammaproteobacteria* cells, while bidirectional arrow in T highlights bacteria cells stained by the universal probe EUB338MIX only); first column panels (**A**, **E**, **I**, **M** and **Q**) show the autofluorescence of the seed tissues. Scale bars: 20 μm (all panels). *pa* parenchyma, *tg* tegument or seed coat (mesotesta), *vt* vascular tissues, *b* bacteria, *g* *Gammaproteobacteria*.

**Figure 5:**
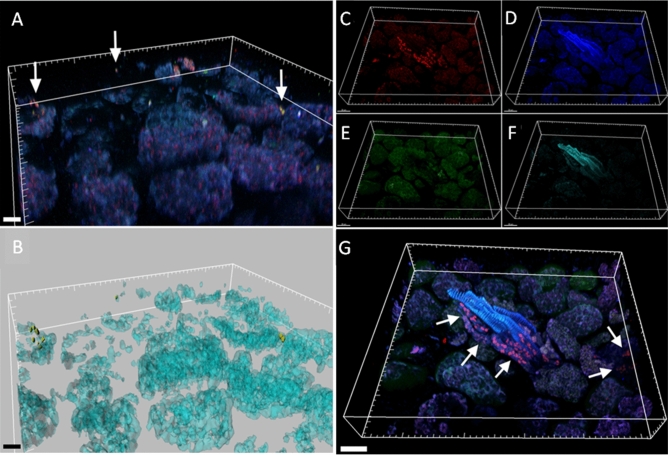
Bacterial colonization of *C. limon* shoots regenerated in aseptic condition from surface-sterilized seeds. Confocal laser scanning microscopy images showing the bacterial colonization of *Citrus limon* (L.) Burm. F. in vascular bundles and parenchyma of shoots germinated by surface-sterilized seeds. Cryosections were stained by Fluorescent In Situ Hybridization. (**A**) Volume renderings showing *Firmicutes* bacteria (signal of the *Firmicutes*-specific probe LGC354MIX, arrows). (**B**) Three-dimensional model of panel (**A**). (**C**) Volume renderings showing unidentified bacteria inside the vascular bundles (signal of the EUB338MIX universal probe, arrows). (**D**–**F**), Volume renderings showing shoot tissue auto-fluorescence; (**G**) overlap of panels (**C**–**F**). Scale bars = 20 µm.

Surface-sterilized seed and sections of shoots regenerated under aseptic conditions from surface-sterilized seed of *C. limon*, were analyzed by FISH-CLSM. Universal and phylum/class-specific probes (i.e. *Firmicutes*, *Gammaprtoeobacteria* and *Betaproteobacteria*) were used to localize endophytic bacteria in the different sites. A relatively few number of endophytes was revealed in seeds. In particular in the parenchyma (Fig. 4A–L) and tegument seed sections (Fig. 4 M–P), unclassified bacteria (red signals) and bacteria belonging to *Firmicutes* and *Gammaproteobacteria* (yellow signals) were detected. Interestingly, most of the bacterial cells were mainly located in the vascular tissues (Fig. 4Q–T). Bacteria were also detected in the stem tissues of shoot, both in the intercellular spaces (*Firmicutes*) or above cells (Fig. 5A,B, arrows), and associated to vascular bundles, as revealed by a 3D reconstruction (Fig. 5 C-G, arrows). Staining with NONEUB non-sense probe provided complete absence of probe-conferred signals in the same tissues (Supplementary Fig. S5), confirming the specificity of bacterial probe signals.

## Discussion and conclusions

The importance of the microbiota for plant fitness has been widely recognized. This led to the concept of “plant holobiont”^1^, where plants and microorganisms are associated, forming an interkingdom community of species, assembled around the host plant. Seeds are considered the intergenerational reservoirs and vectors for beneficial and sometimes detrimental microorganisms. For many years the role of plant seeds in shaping and shuttling complex endophyte community has been overlooked. In this study, fungal and bacterial endophyte communities residing in *C. limon* seeds were characterized through a polyphasic approach including strain isolation coupled with phylogentic classification and NGS of 16S rRNA gene V4 region and fungal ITS2 amplicons from metagenomic DNA. The localization of bacterial endophytes in *C. limon* seed tissues was assessed by FISH-CLSM that highlighted a relatively few number of endophytes specifically localized, particularly, in proximity of vascular bundles. Finally and noteworthy, shoots regenerated from surface sterilized seeds in aseptic conditions showed a microbiota whose structure and distribution maintain analogies with seed endophyte microbiota, as demonstrated by NGS analysis of 16S rRNA gene V4 and fungal ITS2 region and FISH-CLSM investigations.

The isolated bacteria belong to the *Staphylococcus* genus. In particular, they are phylogenetically related to the *epidermidis*, *hominis* and *warneri* species, respectively. Staphylococci, generally considered typical human-associated bacteria with pathogenic potential, were often revealed in association with plants. In particular, they were found as seed endophytes, as examples, of *A. colubrina*^5^ and rice^42^; in addition, they were isolated from different tissues of plants such as soybean^43^. Regarding the fungal isolates, *Aspergillus* is one of the most widespread and ubiquitous fungal genus, *Quambalaria* and *Bjerkandera* are a genera whose members are often associated with plants including *Citrus* spp. It is interesting to note that strains belonging to *Aspergillus* genus are often isolated from *Citrus* sp. fruits^44^; indeed, they are one of the most used fungal genus to process *Citrus* sp. derived food and waste^45,46^ and they are often used for the production of citric acid at the industrial level^47^. Thus, this suggests a strong adaptive evolution of members of this genus to use *Citrus* sp. derived macromolecules.

Two *Staphylococcus* and one *Aspergillus* isolates were able to synthetize IAA and all the isolates were able to grow on different phosphate sources (Table 1). In addition, some of them produced diffusible factors solubilizing phosphate, as it was inferred from the formation of solubilization halos (Table 1)^18,48^. Thus, these results indicate a potential role as plant growth promoting microorganisms.

In this work, the isolated strains do not belong to the most abundant genera revealed by NGS analysis. The discrepancy between culture-dependent and NGS results is usually expected since, as previously reported^49^, the two approaches rely on different criteria to reveal microorganisms. Indeed, even using different approaches—including colony picking from agar plates, dilution in liquid media in 96-well microtitre plates or bacterial cell sorting—and also using different growth media, microbial cultivations cannot deliver a total coverage of the whole microbiota but, nevertheless, it could achieve a satisfactory representation^50,51^. Anyhow, by culture dependent approaches it is possible to isolate microbial strains even under the limit of detection of metagenomic approaches and/or 16S rRNA gene sequencing. Therefore, the two approaches can be complementary, being NGS-based analysis usually more comprehensive but not always more sensible than culture-dependent methods^51^.

Reads from NGS analysis that have been successfully identified and derived from microbial DNA, were a minority with the respect to those derived from plant DNA, especially in the case of fungal endophytes. Retrieving few microbial sequences in the seeds could be expected likely to be due to the low amount of microbial DNA in comparison to the abundant plant genomes. Indeed, other works, dealing with NGS-based characterization of seed endophytic microbiota, report a number of microbial reads ranging from a few hundred or a few thousand to tens of thousands^52,53^, in such a way that could be related with the real number of microbiota members. Experimental bias in microbiota characterization based on NGS analysis of 16S rRNA genes and ITS sequences cannot be completely excluded^54^, especially in the case of the low number of reads like in the case of *C. limon* seed fungal microbiota. Anyhow, taking into account the results of sterility controls (Supplementary Fig. S1), from one hand, and the similarity between seed and shoot microbiota as inferred from beta diversity (Supplementary Table S8) and PERMANOVA (Supplementary Table S9) values, from the other one, our results could be considered representative of the microbiota composition of the *C. limon* seeds and shoots.

The low amount of bacterial cells in comparison to plant cells is also supported by the FISH-based investigation here shown. Indeed, microscopic analysis of colonization pattern is a critical aspect when investigating natural host-associated microbial communities to confirm molecular and culture-dependent results^55,56^. In the present work, bacterial niches of colonization in the seed endosphere were highlighted by FISH staining, confirming the presence of the taxa (*Firmicutes*) detected by isolation, but also highlighted the occurrence of additional taxa such as *Gammaproteobacteria*, coherently with the NGS-based analysis. Interestingly, bacterial cells were revealed in the intercellular spaces of the seed tegument, parenchyma and vascular tissues in seeds, as previously reported^5^, but also in shoots. The detection of bacterial cells in the intercellular spaces is coherent with the typical colonization pattern of plant endophytes, which usually colonize apoplastic spaces^56^. Intriguingly, the localization in the vascular tissues supports the possible way of vertical transmission and internal translocation of endophytes from seed to shoot.

Alpha-diversity indices (Supplementary Table S7) highlighted that the microbial diversity increased upon seed germination for the bacterial component while decreased for the fungal counterpart of the community. The results suggest that bacterial rare species were able to thrive increasing their relative abundance. On the contrary, the major members of fungal community of *C. limon* seeds became more and more abundant. However, as above mentioned, beta diversity indices (Supplementary Table S8) suggest that bacterial and fungal community are quite similar between seeds and shoots as also confirmed by PERMANOVA values (Supplementary Table S9). Indeed, this is consistent with the fact that the major representative OTUs are in common since about 50% of reads are shared between the two tissues (Supplementary Fig. S4).

In general, most bacterial and fungal genera of *C. limon* seed microbiota have already been identified as microbiota components of different plants, including *Citrus* species. Also novel genera appear as seed endophytes. Interestingly, *Pseudomonas*,* Rhodococcus* and *Chryseobacterium* bacterial genera and *Aspergillus*, *Penicillium*, *Exophiala* and *Fusarium* fungal genera are present in the microbiota of *C. limon* seeds and shoots thereof and in the microbiota of *C. limon* root and ryzosphere thereof^57^, thus, suggesting the existence of a *C. limon* core microbiota that is not dependent by plant growth or experimental conditions.

In addition, as it was discussed in the case of *Staphylococcus* isolates, also some of the NGS identified genera comprise important members of human microbiota. As an example, *Cutibacterium* genus members, that are present in both *C. limon* seeds and in shoots, have been reported either as endophytes in the domesticated grapevine *Vitis vinifera* and like members of human skin microbiota. Interestingly, if the possible contaminations due to experimental procedures could be completely excluded, altogether those evidences would suggest that the sharing of bacteria belonging to the human skin microbiota with domesticated plant endophytes might be a wider phenomenon than previously expected^58^ and this point would deserve dedicated investigations. In addition, many bacterial genera that were identified as members of *C. limon* seed and/or shoot microbiota are known to exert PGP activities. For instance, both seeds and shoots contain *Acinetobacter* genus members, that have been already revealed in the rhizosphere as IAA producers, able to solubilize phosphates producing siderophores and capable to prevent the spread of phytopathogens^59^. In addition, the members of many fungal genera among *C. limon* seed endophytes are often reported as associated to roots, even in mycorrhizal structures. It is fascinating to hypothesize that seed associated fungi might help the germination process and the development of rooting system in *Citrus*, as previously reported in other systems^60^. A detailed description of possible biological roles of major microbial taxa identified by NGS analysis is provided as Supplementary Materials.

A combined approach based on culture-dependent assays, NGS-based approaches and FISH-CLSM microscopy, was performed to reveal the endophytic bacterial and fungal microbiota of *Citrus limon* seeds. This investigation also revealed transmission of microbiota members to post-embryonic development tissues of shoots, thus corroborating the idea of a vertical transmission of plant microbiota in *C. limon*. The possible plant growth promoting activities, inferred from experimental data or based on scientific literature concerning related microbial genera, suggest a beneficial role in seed germination and plant growth of endophytic bacterial and fungal microbiota. On the other hand, it could be interesting to evaluate the effect of dysbiosis, at level of seed microbiota, on germination and plant development. In this context, complementary approaches, such as nested PCR, could be a useful technique to investigate the presence of poorly represented strains that, despite their abundance, can play a crucial biological role on determining plant health.

Therefore, the understanding of the composition and structure of the seed-associated microbiome of *Citrus* can have weighty impacts on the germinating seeds themselves, on growing seedlings and on adult plants. Thus, in perspective, this knowledge could have consequences either in the full understanding of plant biology, and in biotechnological applications, aimed at developing effective predictive tools for plant health and/or bio-fertilizers for sustainable agriculture.

## Supplementary Information

Supplementary Information
