# Supplementary material for: The endophytic microbiota of Citrus limon is transmitted from seed to shoot highlighting differences of bacterial and fungal community structures
Source: Sci Rep. 2021 Mar 29;11:7078. doi: 10.1038/s41598-021-86399-5 (PMC8007603; doi:10.1038/s41598-021-86399-5)
Supplement: Supplementary file 1 — Supplementary Information [file 41598_2021_86399_MOESM1_ESM.docx]

**Supplementary materials**

**The endophytic microbiota of *Citrus limon* is transmitted from seed to shoot highlighting differences of bacterial and fungal community structures**

Teresa Faddetta^1#^, Loredana Abbate^2#^, Pasquale Alibrandi^3^, Walter Arancio^1,4^, Davide Siino^1^, Francesco Strati^5^, Carlotta De Filippo^6^, Sergio Fatta Del Bosco^2^, Francesco Carimi^2^, Anna Maria Puglia^1^, Massimiliano Cardinale^7,8^, Giuseppe Gallo^1^*, Francesco Mercati^2^

*^1^Department of Biological, Chemical and Pharmaceutical Sciences and Technologies (STEBICEF), University of Palermo, Palermo, Italy*

*^2^Institute of Biosciences and Bioresources (IBBR), National Research Council, Palermo, Italy*

*^3^ Department of Life Sciences and Systems Biology, University of Turin, Italy*

*^4^Ri.MED Foundation, Palermo, Italy*

*^5^Laboratory of Mucosal Immunology, Department of Experimental Oncology, European Institute of Oncology, Milano, Italy*

*^6^Institute of Agricultural Biology and Biotechnology, National Research Council, Pisa, Italy*

*^7^Institute of Applied Microbiology, Justus-Liebig-University Giessen, Giessen, Germany*

*^8^Department of Biological and Environmental Sciences and Technologies (DiSTeBA), University of Salento, Lecce, Italy*

^#^The authors contributed equally to this work.

*corresponding author: Dr. Giuseppe Gallo, giuseppe.gallo@unipa.it

**Focus on microbial endophytic community structure of *C. limon* seeds and shoots**

In seeds, two unclassified bacterial genera, belonging to *Sphingomonadaceae* and *Burkholderiaceae* families, are the most abundant ones accounting for 22.3 % and 15.6 % of total reads, respectively. Members of *Sphingomonadaceae* family have been previously reported as abundant in plant species, such as in *Medicago sativa* ^1^, as well as *Burkholderiaceae* family members showed to exert beneficial effects in *Solanum lycopersicum* L. and *Brassica napus* ^2^. In the shoot tissues their relative abundance decreases and most prominent genus is the above mentioned *Cutibacterium*. Other bacteria characterizing shoot tissues are: *Emticicia* (4.8 % of total reads), previously associated to mosses ^3^ but whose possible role is to be elucidated yet; *Porphyrobacter* (4.2%), belonging to photosynthesizing bacteria, isolated in cyanobacteria consortia ^4^ but, at the best of our knowledge, never before been associated to plants; *Arenimonas* (3.6 %*)*, genus comprising *A. oryziterrae* that has been isolated from rice ^5^; *Pseudomonas* (3.1%), whose presence may be related to the well-studied plant-bacteria interactions ^6^, especially at the level of rhizosphere ^7^.

Fungal microbiota in seeds is enriched in the *Lophiostomataceae* family (20.7%), whose members are distributed especially in temperate regions, consisting of saprobic or necrotrophic members, found typically on herbaceous and woody stems. *Cladosporium* spp. (11,2%) are ubiquitous worldwide: they are quite common on living and dead plant material and it has been already reported on *Citrus sinensis* leaves ^8^; in addition, some species are plant pathogens, while other species have been reported as promoter of plant growth via competition with pathogens ^9^. Interestingly, *Cladosporium* spp. have been identified as dark septate endophytes in the roots of ‘non-mycorrhizal’ plants ^10^. Their biological significance is not clear, but their presence in the seed might suggest a positive influence in the root development in the seedlings. *Peniophoraceae* family (11.2 %) is widely distributed and is mostly saprobic. Noteworthy, some authors reported that they are part of mycorrhizal samples ^11^. The 6 % of seed endophytic fungi has been classified as *Xylodon erastii*. Unluckily, the information about the physiology of this species and its interactions with plants are unknown. Instead, 5.6 % of seed endophytic fungi has been assigned to *Itersonilia perplexans* that is reported to be a plant pathogen, causing flower and seedling blight, leaf necrosis, and root cankers in edible roots. Nearly five percent of reads was not able to be classified deeper that *Malasseziales* order. Nevertheless, this order is worth citing because it is usually associated to the skin microbiota, often with pathogenic activity. Indeed, some species of this order have been reported as associated to plant roots (*e.g.* *Festuca paniculata* in subalpine grasslands ^12^ and *Vaccinium carlesii* in subtropical forests of China ^13^). Additionally, even if the classification of *Heliotales* order (accounting for 5.2 % of total reads of seed microbiota) failed to discriminate at subsequent classification levels, it is worth citing because *Heliotales* fungi show a widely reported interaction with plants: they span from pathogenic, saprotrophic and mycoparasitic activities to endophytic, mycorhizzal and symbiotic activities on roots ^14^. Information about *Sistotrema coronilla* and *Burgoa verzuoliana* (3.5 % and 3.0 %, respectively) is rare. It is known that *B. verzuolana* is associated to lichen systems ^15^, but never an involvement of either has been reported in seed development or functions. The other classified fungal OTUs account each for less than 3 % of total reads in seeds. Concerning the shoots obtained in aseptic condition from surface-sterilized seeds, *Capnodiales* order represents the 35.8 % of fungal reads. *Capnodiales* order comprehends species with a very diverse behavior. They range from plant and animal pathogens, to endophytes and epiphytes, and even many species are lichenized ^16^. Noteworthy, the *Cladosporium* genus, isolated in seeds, is part of the *Capnodiales* order. *Debaryomyces hansenii* (17.4 %) is an interesting species to be found in shoot. It is a salt tolerant yeast very common in processed cheese and food ^17^, known for the production of mycocins, toxins able to destroy competitive yeast species ^18^. Its conspicuous presence in embryos can be interpreted either as another example of the effect of domestication and/or as protective against other form of pathogenic fungi. The genus *Penicillium* (10.7 %) is of major importance in the natural environment, in food processing and drug industries. The positive effects of *Penicillium* spp. on plant growth are well known and documented, by the way of phosphorous mobilization ^19^, protection from root disease ^20^, and generally in maintaining soil health by phosphate solubilization, production of siderophore, and production of diffusible plant-growth regulators ^21^. *Candida intermedia* (5.4 %) has been reported as a species able to exert killer activities on phytopathogenic molds in plats of agriculture interest ^22^. *Filobasidium stepposum* (4.9%) has been reported as part of spring seasonal fungal consortia in Italian soils ^23^. Noteworthy, *Lophiostomataceae* family and *Pseudotomentella tristis* have been identified also in shoot tissues (4.7% and 2.1%, respectively) and not only in seeds, suggesting, together with diversity indices discussed above, that upon germination, part of the seed associated microbiota could thrive and concur to the development of the shoot-associated microbiota. The other classified fungal OTUs account each for less than 3 % of total reads.

**References**

1. Pini, F. *et al.* Exploring the plant-associated bacterial communities in *Medicago sativa* L. *BMC Microbiol.* **12**, 78 (2012).

2. Rybakova, D. *et al.* The structure of the *Brassica napus* seed microbiome is cultivar-dependent and affects the interactions of symbionts and pathogens. *Microbiome* **5**, 104 (2017).

3. Tian, Y. & Li, Y. H. Comparative analysis of bacteria associated with different mosses by 16S rRNA and 16S rDNA sequencing. *J. Basic Microbiol.* **57**, 57–67 (2017).

4. Hughes, R.-A. *et al.* Genome sequence, metabolic properties and cyanobacterial attachment of *Porphyrobacter* sp. HT-58-2 isolated from a filamentous cyanobacterium–microbial consortium. *Microbiology* **164**, 1229–1239 (2018).

5. Aslam, Z., Park, J. H., Kim, S. W., Jeon, C. O. & Chung, Y. R. *Arenimonas oryziterrae* sp. nov., isolated from a field of rice (*Oryza sativa* L.) managed under a no-tillage regime, and reclassification of *Aspromonas composti* as *Arenimonas composti* comb. nov. *Int. J. Syst. Evol. Microbiol.* **59**, 2967–2972 (2009).

6. Sitaraman, R. *Pseudomonas* spp. as models for plant-microbe interactions. *Frontiers in Plant Science*  **6**, 787 (2015).

7. Lundberg, D. S. *et al.* Defining the core *Arabidopsis thaliana* root microbiome. *Nature* **488**, 86–90 (2012).

8. Bensch, K. *et al.* Species and ecological diversity within the *Cladosporium cladosporioides* complex (*Davidiellaceae*, *Capnodiales*). *Stud. Mycol.* **67**, 1–94 (2010).

9. Torres, D. E. *et al.* *Cladosporium* *cladosporioides* and *Cladosporium pseudocladosporioides* as potential new fungal antagonists of P*uccinia horiana* Henn., the causal agent of chrysanthemum white rust. *PLoS One* **12**, 1–16 (2017).

10. Liu, H., Li, T., Ding, Y., Yang, Y. & Zhao, Z. Dark septate endophytes colonizing the roots of ‘non-mycorrhizal’ plants in a mine tailing pond and in a relatively undisturbed environment, Southwest China. *J. Plant Interact.* **12**, 264–271 (2017).

11. Kinoshita, A. *et al.* How do fungal partners affect the evolution and habitat preferences of mycoheterotrophic plants? A case study in Gastrodia. *Am. J. Bot.* **103**, 207–220 (2016).

12. Mouhamadou, B. *et al.* Differences in fungal communities associated to *Festuca paniculata* roots in subalpine grasslands. *Fungal Divers.* **47**, 55–63 (2011).

13. Zhang, Y. *et al.* Root-associated fungi of *Vaccinium carlesii* in subtropical forests of China: intra-and inter-annual variability and impacts of human disturbances. *Sci. Rep.* **6**, 1–12 (2016).

14. van der Heijden, M. G. A., Dombrowski, N. & Schlaeppi, K. Continuum of root-fungal symbioses for plant nutrition. *Proc. Natl. Acad. Sci. U. S. A.* **114**, 11574–11576 (2017).

15. Lawrey, J. D. *et al.* Phylogenetic diversity of lichen-associated homobasidiomycetes. *Mol. Phylogenet. Evol.* **44**, 778—789 (2007).

16. Crous, P. W. *et al.* Phylogenetic lineages in the Capnodiales. *Stud. Mycol.* **64**, 17–47 (2009).

17. Banjara, N., Suhr, M. J. & Hallen-Adams, H. E. Diversity of yeast and mold species from a variety of cheese types. *Curr. Microbiol.* **70**, 792–800 (2015).

18. Banjara, N., Nickerson, K. W., Suhr, M. J. & Hallen-Adams, H. E. Killer toxin from several food-derived *Debaryomyces hansenii* strains effective against pathogenic *Candida* yeasts. *Int. J. Food Microbiol.* **222**, 23–29 (2016).

19. Wakelin, S. A., Gupta, V. V. S. R., Harvey, P. R. & Ryder, M. H. The effect of *Penicillium* fungi on plant growth and phosphorus mobilization in neutral to alkaline soils from southern Australia. *Can. J. Microbiol.* **53**, 106–115 (2007).

20. Radhakrishnan, R., Kang, S.-M., Baek, I.-Y. & Lee, I.-J. Characterization of plant growth-promoting traits of *Penicillium* species against the effects of high soil salinity and root disease. *J. Plant Interact.* **9**, 754–762 (2014).

21. Altaf, M. M., Imran, M., Abulreesh, H. H., Khan, M. S. A. & Ahmad, I. Diversity and applications of *Penicillium* spp. in plant-growth promotion. *New Futur. Dev. Microb. Biotechnol. Bioeng.* 261–276 (2018). doi:10.1016/B978-0-444-63501-3.00015-6

22. Rosa-Magri, M. M., Tauk-Tornisielo, S. M. & Ceccato-Antonini, S. R. Bioprospection of yeasts as biocontrol agents against phytopathogenic molds. *Brazilian Arch. Biol. Technol.* **54**, 1–5 (2011).

23. Panelli, S. *et al.* A metagenomic-based, cross-seasonal picture of fungal consortia associated with Italian soils subjected to different agricultural managements. *Fungal Ecol.* **30**, 1–9 (2017).

**Figure S1 Scheme of experimental procedures to obtain surface-sterilized *C. limon* seeds.** Cultivable endophytes were obtained after homogenization of surface-sterilized seeds in step 4. Sterility of seed surface was assessed by plating washing water obtained in step 2.

**Figure S2 Workflow describing the procedures performed to obtain metagenomic DNA from *C. limon* surface-sterilized seeds and from shoots obtained from *C. limon* surface-sterilized seeds.**

**Figure S3. Venn diagram describing shared OTUs between surface-sterilized seeds and shoots regenerated under aseptic conditions from surface-sterilized seeds.** A) The number of bacterial OTUs from shoots is 136, and the number of sequences is 3907; 1687 sequences are not shared. The number of bacterial OTUs in seed is 99 and the number of sequences is 2386; 1581 sequences are not shared. The number of bacterial shared OTUs is 35 and the number of sequences is 3025; 48.07% of these sequences are shared. Percentage of OTUs shared is 17.50. The total richness is 200. B) The number of fungal OTUs in shoots is 36 and the number of sequences is 2775; 1484 sequences are not shared. The number of OTUs in seed is 20, and the number of sequences is 232; 141 sequences are not shared. The number of OTUs shared between groups is 7, and the number of sequences is 1382; 45.96% of these sequences are shared. Percentage of OTUs shared is 14,29. The total richness is 49.

**Figure S4. Rarefaction curves showing the observed OTU richness at 97 % identity.** A) 16S rDNA amplicon libraries. B) fungal ITS region of small-subunit rDNA amplicons.

**Figure S5. Negative control of FISH staining.** A subsample of shoot tissue was stained with the non-sense FISH probe NON-EUB, and revealed no probe-conferred signal (compare with Fig. 6 C-G). Scale bars= 20 µm.

**Table S1. Identification of isolated bacterial strains by SILVA “Alignment, Classification and Tree” Service analysis.**

| **Sequence identifier** | **lca_tax_slv** | **Sequence score** | **Bp score** | **identity** |
| --- | --- | --- | --- | --- |
| B1 | *Staphylococcus* | 0.998 | 105 | 999.293 |
| B2 | *Staphylococcus* | 0.999 | 104 | 999.287 |
| B3 | *Staphylococcus* | 0.991 | 108 | 994.398 |

**Table S2. Identification of isolated fungal strains by UNITE nBLAST analysis.**

| **Query** | **Reference sequence** | **Score** | **E-value** |
| --- | --- | --- | --- |
| F1 | KX463362\|SH1572953.08FU\|*Aspergillus pseudoglaucus* | 1053 | 0.0 |
| F2 | KX674666\|SH1552035.08FU\|*Quambalaria cyanescens* | 1236 | 0.0 |
| F3 | KX894661\|SH1549900.08FU\|*Aspergillus protuberus* | 1064 | 0.0 |
| F4 | JF721422\|SH1615228.08FU\|*Bjerkandera* | 1179 | 0.0 |

| **Table S3. Taxonomical classification and abundance of bacterial microbiota members based on 16S rDNA V4 sequences obtained by NGS analysis of total DNA extracted from surface-sterilized *C. limon* seeds.** Confidence values are enclosed between parentheses. | | | | | | | |
| --- | --- | --- | --- | --- | --- | --- | --- |
| **KINGDOM** | **PHYLUM** | **CLASS** | **ORDER** | **FAMILY** | **GENUS** | **NUMBER OF READS** | **PERCENTAGE** |
| Bacteria(100) | Actinobacteria(100) | Actinobacteria(100) | Propionibacteriales(100) | Propionibacteriaceae(100) | Cutibacterium(100) | 314 | 13.160 |
| Bacteria(100) | Actinobacteria(100) | Actinobacteria(100) | Streptomycetales(100) | Streptomycetaceae(100) | Streptomyces(99) | 53 | 2.221 |
| Bacteria(100) | Actinobacteria(100) | Thermoleophilia(100) | Solirubrobacterales(100) | 67-14(100) | 67-14_ge(100) | 32 | 1.341 |
| Bacteria(100) | Actinobacteria(100) | Actinobacteria(100) | Corynebacteriales(100) | Nocardiaceae(100) | Rhodococcus(100) | 11 | 0.461 |
| Bacteria(100) | Actinobacteria(100) | Actinobacteria(100) | Corynebacteriales(100) | Corynebacteriaceae(100) | Lawsonella(100) | 10 | 0.419 |
| Bacteria(100) | Actinobacteria(100) | Actinobacteria(100) | Corynebacteriales(100) | Mycobacteriaceae(100) | Mycobacterium(100) | 6 | 0.251 |
| Bacteria(100) | Actinobacteria(100) | Actinobacteria(100) | Corynebacteriales(100) | Corynebacteriaceae(100) | Corynebacterium_1(100) | 5 | 0.210 |
| Bacteria(100) | Actinobacteria(100) | Actinobacteria(100) | Propionibacteriales(100) | Propionibacteriaceae(100) | Cutibacterium(100) | 4 | 0.168 |
| Bacteria(100) | Actinobacteria(100) | Actinobacteria(100) | Corynebacteriales(100) | Corynebacteriaceae(100) | Corynebacteriaceae_unclassified(75) | 4 | 0.168 |
| Bacteria(100) | Actinobacteria(100) | Actinobacteria(100) | Propionibacteriales(100) | Propionibacteriaceae(100) | Friedmanniella(100) | 3 | 0.126 |
| Bacteria(100) | Actinobacteria(100) | Actinobacteria(100) | Micrococcales(100) | Micrococcaceae(100) | Micrococcaceae_unclassified(100) | 2 | 0.084 |
| Bacteria(100) | Actinobacteria(100) | Actinobacteria(100) | Micrococcales(100) | Micrococcaceae(100) | Micrococcus(100) | 1 | 0.042 |
| Bacteria(100) | Actinobacteria(100) | Actinobacteria(100) | Corynebacteriales(100) | Nocardiaceae(100) | Nocardia(100) | 1 | 0.042 |
| Bacteria(100) | Actinobacteria(100) | Actinobacteria(100) | Frankiales(100) | Geodermatophilaceae(100) | Geodermatophilus(100) | 1 | 0.042 |
| Bacteria(100) | Actinobacteria(100) | Actinobacteria(100) | Corynebacteriales(100) | Corynebacteriaceae(100) | Lawsonella(100) | 1 | 0.042 |
| Bacteria(100) | Actinobacteria(100) | Actinobacteria(100) | Actinobacteria_unclassified(100) | Actinobacteria_unclassified(100) | Actinobacteria_unclassified(100) | 1 | 0.042 |
| Bacteria(100) | Bacteria_unclassified(100) | Bacteria_unclassified(100) | Bacteria_unclassified(100) | Bacteria_unclassified(100) | Bacteria_unclassified(100) | 2 | 0.084 |
| Bacteria(100) | Bacteroidetes(100) | Bacteroidia(100) | Chitinophagales(100) | Chitinophagaceae(100) | Flavisolibacter(100) | 62 | 2.598 |
| Bacteria(100) | Bacteroidetes(100) | Bacteroidia(100) | Chitinophagales(100) | Chitinophagaceae(100) | Sediminibacterium(100) | 58 | 2.431 |
| Bacteria(100) | Bacteroidetes(100) | Bacteroidia(100) | Chitinophagales(100) | Chitinophagaceae(100) | Lacibacter(100) | 33 | 1.383 |
| Bacteria(100) | Bacteroidetes(100) | Bacteroidia(100) | Bacteroidia_unclassified(100) | Bacteroidia_unclassified(100) | Bacteroidia_unclassified(100) | 29 | 1.215 |
| Bacteria(100) | Bacteroidetes(100) | Bacteroidia(100) | Cytophagales(100) | Microscillaceae(100) | OLB12(100) | 20 | 0.838 |
| Bacteria(100) | Bacteroidetes(100) | Bacteroidia(100) | Chitinophagales(100) | Chitinophagaceae(100) | uncultured(100) | 3 | 0.126 |
| Bacteria(100) | Bacteroidetes(100) | Bacteroidia(100) | Cytophagales(100) | Hymenobacteraceae(100) | Hymenobacter(100) | 2 | 0.084 |
| Bacteria(100) | Bacteroidetes(100) | Bacteroidia(100) | Flavobacteriales(100) | Weeksellaceae(100) | Chryseobacterium(100) | 2 | 0.084 |
| Bacteria(100) | Bacteroidetes(100) | Bacteroidia(100) | Flavobacteriales(100) | Flavobacteriaceae(100) | Flavobacterium(100) | 1 | 0.042 |
| Bacteria(100) | Bacteroidetes(100) | Bacteroidia(100) | Chitinophagales(100) | Chitinophagaceae(100) | Lacibacter(100) | 1 | 0.042 |
| Bacteria(100) | Chloroflexi(100) | Chloroflexia(100) | Thermomicrobiales(100) | JG30-KF-CM45(100) | JG30-KF-CM45_ge(100) | 9 | 0.377 |
| Bacteria(100) | Chloroflexi(100) | Chloroflexia(100) | Thermomicrobiales(100) | JG30-KF-CM45(100) | JG30-KF-CM45_ge(100) | 6 | 0.251 |
| Bacteria(100) | Chloroflexi(100) | Chloroflexia(100) | Thermomicrobiales(100) | JG30-KF-CM45(100) | JG30-KF-CM45_ge(100) | 3 | 0.126 |
| Bacteria(100) | Dadabacteria(100) | Dadabacteriia(100) | Dadabacteriales(100) | Dadabacteriales_fa(100) | Dadabacteriales_ge(100) | 1 | 0.042 |
| Bacteria(100) | Firmicutes(100) | Clostridia(100) | Clostridiales(100) | Ruminococcaceae(100) | Ruminococcaceae_UCG-002(100) | 22 | 0.922 |
| Bacteria(100) | Firmicutes(100) | Bacilli(100) | Bacillales(100) | Staphylococcaceae(100) | Staphylococcus(100) | 20 | 0.838 |
| Bacteria(100) | Firmicutes(100) | Bacilli(100) | Bacillales(100) | Bacillales_unclassified(89) | Bacillales_unclassified(89) | 18 | 0.754 |
| Bacteria(100) | Firmicutes(100) | Clostridia(100) | Clostridiales(100) | Family_XI(100) | Anaerococcus(100) | 7 | 0.293 |
| Bacteria(100) | Firmicutes(100) | Negativicutes(100) | Selenomonadales(100) | Veillonellaceae(100) | Veillonella(100) | 5 | 0.210 |
| Bacteria(100) | Firmicutes(100) | Clostridia(100) | Clostridiales(100) | Lachnospiraceae(100) | Lachnospiraceae_unclassified(100) | 4 | 0.168 |
| Bacteria(100) | Firmicutes(100) | Negativicutes(100) | Selenomonadales(100) | Veillonellaceae(100) | Veillonella(100) | 3 | 0.126 |
| Bacteria(100) | Firmicutes(100) | Bacilli(100) | Lactobacillales(100) | Carnobacteriaceae(100) | Granulicatella(100) | 3 | 0.126 |
| Bacteria(100) | Firmicutes(100) | Bacilli(100) | Bacillales(100) | Paenibacillaceae(100) | Brevibacillus(100) | 2 | 0.084 |
| Bacteria(100) | Firmicutes(100) | Bacilli(100) | Lactobacillales(100) | Lactobacillaceae(100) | Lactobacillus(100) | 2 | 0.084 |
| Bacteria(100) | Firmicutes(100) | Bacilli(100) | Bacillales(100) | Bacillaceae(100) | Bacillaceae_unclassified(100) | 1 | 0.042 |
| Bacteria(100) | Firmicutes(100) | Bacilli(100) | Lactobacillales(100) | Aerococcaceae(100) | Abiotrophia(100) | 1 | 0.042 |
| Bacteria(100) | Gemmatimonadetes(100) | Gemmatimonadetes(100) | Gemmatimonadales(100) | Gemmatimonadaceae(100) | Gemmatimonadaceae_unclassified(100) | 64 | 2.682 |
| Bacteria(100) | Planctomycetes(100) | Planctomycetacia(100) | Planctomycetales(100) | uncultured(100) | uncultured_ge(100) | 2 | 0.084 |
| Bacteria(100) | Planctomycetes(100) | Planctomycetacia(100) | Isosphaerales(100) | Isosphaeraceae(100) | Isosphaera(100) | 1 | 0.042 |
| Bacteria(100) | Planctomycetes(100) | Planctomycetacia(100) | Planctomycetales(100) | uncultured(100) | uncultured_ge(100) | 1 | 0.042 |
| Bacteria(100) | Proteobacteria(100) | Alphaproteobacteria(100) | Sphingomonadales(100) | Sphingomonadaceae(100) | Sphingomonadaceae_unclassified(100) | 532 | 22.297 |
| Bacteria(100) | Proteobacteria(100) | Gammaproteobacteria(100) | Betaproteobacteriales(100) | Burkholderiaceae(100) | Burkholderiaceae_unclassified(100) | 372 | 15.591 |
| Bacteria(100) | Proteobacteria(100) | Gammaproteobacteria(100) | Pseudomonadales(100) | Moraxellaceae(100) | Acinetobacter(100) | 193 | 8.089 |
| Bacteria(100) | Proteobacteria(100) | Alphaproteobacteria(100) | Sphingomonadales(100) | Sphingomonadaceae(100) | Sphingopyxis(100) | 73 | 3.060 |
| Bacteria(100) | Proteobacteria(100) | Gammaproteobacteria(100) | Betaproteobacteriales(100) | Burkholderiaceae(100) | Burkholderiaceae_unclassified(89) | 36 | 1.509 |
| Bacteria(100) | Proteobacteria(100) | Gammaproteobacteria(100) | Xanthomonadales(100) | Xanthomonadaceae(100) | Vulcaniibacterium(100) | 36 | 1.509 |
| Bacteria(100) | Proteobacteria(100) | Gammaproteobacteria(100) | Enterobacteriales(100) | Enterobacteriaceae(100) | Escherichia-Shigella(73) | 33 | 1.383 |
| Bacteria(100) | Proteobacteria(100) | Gammaproteobacteria(100) | Pseudomonadales(100) | Pseudomonadaceae(100) | Pseudomonas(97) | 30 | 1.257 |
| Bacteria(100) | Proteobacteria(100) | Alphaproteobacteria(100) | Rhizobiales(100) | Rhizobiaceae(100) | Rhizobiaceae_unclassified(100) | 21 | 0.880 |
| Bacteria(100) | Proteobacteria(100) | Alphaproteobacteria(100) | Rhizobiales(100) | Rhizobiaceae(100) | Rhizobiaceae_unclassified(100) | 20 | 0.838 |
| Bacteria(100) | Proteobacteria(100) | Gammaproteobacteria(100) | Alteromonadales(100) | Alteromonadaceae(100) | Rheinheimera(100) | 19 | 0.796 |
| Bacteria(100) | Proteobacteria(100) | Deltaproteobacteria(100) | Myxococcales(100) | Myxococcales_unclassified(100) | Myxococcales_unclassified(100) | 19 | 0.796 |
| Bacteria(100) | Proteobacteria(100) | Gammaproteobacteria(100) | Vibrionales(100) | Vibrionaceae(100) | Vibrio(75) | 16 | 0.671 |
| Bacteria(100) | Proteobacteria(100) | Gammaproteobacteria(100) | Betaproteobacteriales(100) | Neisseriaceae(100) | uncultured(100) | 16 | 0.671 |
| Bacteria(100) | Proteobacteria(100) | Gammaproteobacteria(100) | Betaproteobacteriales(100) | Burkholderiaceae(100) | Herbaspirillum(100) | 11 | 0.461 |
| Bacteria(100) | Proteobacteria(100) | Gammaproteobacteria(100) | Alteromonadales(100) | Alteromonadaceae(100) | Rheinheimera(100) | 10 | 0.419 |
| Bacteria(100) | Proteobacteria(100) | Proteobacteria_unclassified(100) | Proteobacteria_unclassified(100) | Proteobacteria_unclassified(100) | Proteobacteria_unclassified(100) | 8 | 0.335 |
| Bacteria(100) | Proteobacteria(100) | Gammaproteobacteria(100) | Gammaproteobacteria_unclassified(100) | Gammaproteobacteria_unclassified(100) | Gammaproteobacteria_unclassified(100) | 8 | 0.335 |
| Bacteria(100) | Proteobacteria(100) | Gammaproteobacteria(100) | Pseudomonadales(100) | Moraxellaceae(100) | Psychrobacter(100) | 7 | 0.293 |
| Bacteria(100) | Proteobacteria(100) | Alphaproteobacteria(100) | Rhodobacterales(100) | Rhodobacteraceae(100) | Rhodobacteraceae_unclassified(100) | 7 | 0.293 |
| Bacteria(100) | Proteobacteria(100) | Alphaproteobacteria(100) | Rhizobiales(100) | Hyphomicrobiaceae(84) | Hyphomicrobiaceae_unclassified(84) | 6 | 0.251 |
| Bacteria(100) | Proteobacteria(100) | Alphaproteobacteria(100) | Rhizobiales(100) | Beijerinckiaceae(100) | Methylobacterium(100) | 6 | 0.251 |
| Bacteria(100) | Proteobacteria(100) | Gammaproteobacteria(100) | Xanthomonadales(100) | Xanthomonadaceae(100) | Stenotrophomonas(100) | 5 | 0.210 |
| Bacteria(100) | Proteobacteria(100) | Alphaproteobacteria(100) | Sphingomonadales(100) | Sphingomonadaceae(100) | Sphingomonadaceae_unclassified(100) | 5 | 0.210 |
| Bacteria(100) | Proteobacteria(100) | Alphaproteobacteria(100) | Reyranellales(100) | Reyranellaceae(100) | Reyranella(100) | 5 | 0.210 |
| Bacteria(100) | Proteobacteria(100) | Gammaproteobacteria(100) | Betaproteobacteriales(100) | Nitrosomonadaceae(100) | DSSD61(100) | 5 | 0.210 |
| Bacteria(100) | Proteobacteria(100) | Gammaproteobacteria(100) | Pseudomonadales(100) | Moraxellaceae(100) | Enhydrobacter(100) | 4 | 0.168 |
| Bacteria(100) | Proteobacteria(100) | Gammaproteobacteria(100) | Aeromonadales(100) | Aeromonadaceae(100) | Aeromonas(100) | 4 | 0.168 |
| Bacteria(100) | Proteobacteria(100) | Alphaproteobacteria(100) | Sphingomonadales(100) | Sphingomonadaceae(100) | Sphingomonadaceae_unclassified(100) | 4 | 0.168 |
| Bacteria(100) | Proteobacteria(100) | Deltaproteobacteria(100) | Myxococcales(100) | mle1-27(100) | mle1-27_ge(100) | 4 | 0.168 |
| Bacteria(100) | Proteobacteria(100) | Alphaproteobacteria(100) | Sphingomonadales(100) | Sphingomonadaceae(100) | Sphingomonadaceae_unclassified(100) | 3 | 0.126 |
| Bacteria(100) | Proteobacteria(100) | Gammaproteobacteria(100) | Pseudomonadales(100) | Moraxellaceae(100) | Acinetobacter(100) | 3 | 0.126 |
| Bacteria(100) | Proteobacteria(100) | Gammaproteobacteria(100) | Xanthomonadales(100) | Xanthomonadaceae(100) | Stenotrophomonas(100) | 3 | 0.126 |
| Bacteria(100) | Proteobacteria(100) | Gammaproteobacteria(100) | Gammaproteobacteria_unclassified(100) | Gammaproteobacteria_unclassified(100) | Gammaproteobacteria_unclassified(100) | 3 | 0.126 |
| Bacteria(100) | Proteobacteria(100) | Gammaproteobacteria(100) | Alteromonadales(100) | Alteromonadaceae(100) | Rheinheimera(100) | 3 | 0.126 |
| Bacteria(100) | Proteobacteria(100) | Alphaproteobacteria(100) | Rhodobacterales(100) | Rhodobacteraceae(100) | Rhodobacteraceae_unclassified(100) | 2 | 0.084 |
| Bacteria(100) | Proteobacteria(100) | Gammaproteobacteria(100) | Pseudomonadales(100) | Pseudomonadaceae(100) | Pseudomonas(100) | 2 | 0.084 |
| Bacteria(100) | Proteobacteria(100) | Alphaproteobacteria(100) | Sphingomonadales(100) | Sphingomonadaceae(100) | Sphingomonadaceae_unclassified(100) | 1 | 0.042 |
| Bacteria(100) | Proteobacteria(100) | Gammaproteobacteria(100) | Enterobacteriales(100) | Enterobacteriaceae(100) | Enterobacteriaceae_unclassified(100) | 1 | 0.042 |
| Bacteria(100) | Proteobacteria(100) | Gammaproteobacteria(100) | Pasteurellales(100) | Pasteurellaceae(100) | Pasteurellaceae_unclassified(100) | 1 | 0.042 |
| Bacteria(100) | Proteobacteria(100) | Gammaproteobacteria(100) | Alteromonadales(100) | Shewanellaceae(100) | Shewanella(100) | 1 | 0.042 |
| Bacteria(100) | Proteobacteria(100) | Gammaproteobacteria(100) | Oceanospirillales(100) | Saccharospirillaceae(100) | Bermanella(100) | 1 | 0.042 |
| Bacteria(100) | Proteobacteria(100) | Gammaproteobacteria(100) | Pseudomonadales(100) | Moraxellaceae(100) | Acinetobacter(100) | 1 | 0.042 |
| Bacteria(100) | Proteobacteria(100) | Gammaproteobacteria(100) | Alteromonadales(100) | Pseudoalteromonadaceae(100) | Pseudoalteromonas(100) | 1 | 0.042 |
| Bacteria(100) | Proteobacteria(100) | Alphaproteobacteria(100) | Rhodobacterales(100) | Rhodobacteraceae(100) | Rhodobacteraceae_unclassified(100) | 1 | 0.042 |
| Bacteria(100) | Proteobacteria(100) | Gammaproteobacteria(100) | Betaproteobacteriales(100) | Burkholderiaceae(100) | Burkholderiaceae_unclassified(100) | 1 | 0.042 |
| Bacteria(100) | Proteobacteria(100) | Gammaproteobacteria(100) | Pseudomonadales(100) | Pseudomonadaceae(100) | Pseudomonas(100) | 1 | 0.042 |
| Bacteria(100) | Proteobacteria(100) | Alphaproteobacteria(100) | Sphingomonadales(100) | Sphingomonadaceae(100) | Sphingomonadaceae_unclassified(100) | 1 | 0.042 |
| Bacteria(100) | Proteobacteria(100) | Alphaproteobacteria(100) | Sphingomonadales(100) | Sphingomonadaceae(100) | Sphingomonadaceae_unclassified(100) | 1 | 0.042 |
| Bacteria(100) | Proteobacteria(100) | Gammaproteobacteria(100) | Betaproteobacteriales(100) | Neisseriaceae(100) | Neisseria(100) | 1 | 0.042 |
| Bacteria(100) | Proteobacteria(100) | Alphaproteobacteria(100) | Caulobacterales(100) | Hyphomonadaceae(100) | Hyphomonas(100) | 1 | 0.042 |
| Bacteria(100) | Proteobacteria(100) | Alphaproteobacteria(100) | Rhizobiales(100) | Rhizobiaceae(100) | Rhizobiaceae_unclassified(100) | 1 | 0.042 |

| **Table S4. Taxonomical classification and abundance of fungal microbiota members based on ITS2 sequences obtained by NGS analysis of total DNA extracted from surface-sterilized *C. limon* seeds.** Confidence values are enclosed between parentheses. | | | | | | | |
| --- | --- | --- | --- | --- | --- | --- | --- |
| **KINGDOM** | **PHYLUM** | **CLASS** | **ORDER** | **FAMILY** | **GENUS** | **NUMBER OF READS** | **PERCENTAGE** |
| Fungi (100) | Fungi_unclassified (100) | Fungi_unclassified (100) | Fungi_unclassified (100) | Fungi_unclassified (100) | Fungi_unclassified (100) | 9 | 3.88 |
| Fungi (100) | Fungi_unclassified (100) | Fungi_unclassified (100) | Fungi_unclassified (100) | Fungi_unclassified (100) | Fungi_unclassified (100) | 1 | 0.43 |
| Fungi (100) | Fungi_unclassified (100) | Fungi_unclassified (100) | Fungi_unclassified (100) | Fungi_unclassified (100) | Fungi_unclassified (100) | 1 | 0.43 |
| Fungi (100) | Fungi_unclassified (100) | Fungi_unclassified (100) | Fungi_unclassified (100) | Fungi_unclassified (100) | Fungi_unclassified (100) | 1 | 0.43 |
| Fungi (100) | Basidiomycota (100) | Tremellomycetes (100) | Cystofilobasidiales (100) | Mrakiaceae (100) | Itersonilia (100) | 13 | 5.60 |
| Fungi (100) | Ascomycota (100) | Sordariomycetes (100) | Sordariomycetes_unclassified (100) | Sordariomycetes_unclassified (100) | Sordariomycetes_unclassified (100) | 11 | 4.74 |
| Fungi (100) | Ascomycota (100) | Sordariomycetes (100) | Hypocreales (100) | Stachybotryaceae (100) | Stachybotrys (100) | 5 | 2.16 |
| Fungi (100) | Ascomycota (100) | Sordariomycetes (100) | Saccharomycetales (100) | Dipodascaceae (100) | Yarrowia (100) | 5 | 2.16 |
| Fungi (100) | Basidiomycota (100) | Malasseziomycetes (100) | Malasseziales (100) | unclassified_Malasseziales (100) | unclassified_Malasseziales (100) | 12 | 5.17 |
| Fungi (100) | Ascomycota (100) | Leotiomycetes (100) | Helotiales (100) | Helotiales_unclassified (100) | Helotiales_unclassified (100) | 12 | 5.17 |
| Fungi (100) | Ascomycota (100) | Dothideomycetes (100) | Pleosporales (100) | unclassified_Pleosporales (100) | unclassified_Pleosporales (100) | 8 | 3.45 |
| Fungi (100) | Ascomycota (100) | Dothideomycetes (100) | Pleosporales (100) | Lophiostomataceae (100) | unclassified_Lophiostomataceae (100) | 48 | 20.69 |
| Fungi (100) | Ascomycota (100) | Dothideomycetes (100) | Capnodiales (100) | Cladosporiaceae (100) | Cladosporium (100) | 26 | 11.21 |
| Fungi (100) | Basidiomycota (100) | Agaricomycetes (100) | Thelephorales (100) | Thelephoraceae (100) | Pseudotomentella (100) | 9 | 3.88 |
| Fungi (100) | Basidiomycota (100) | Agaricomycetes (100) | Cantharellales (100) | Cantharellales_fam_Incertae_sedis (100) | Sistotrema (100) | 8 | 3.45 |
| Fungi (100) | Basidiomycota (100) | Agaricomycetes (100) | Polyporales (100) | Polyporales_fam_Incertae_sedis (100) | Burgoa (100) | 7 | 3.02 |
| Fungi (100) | Basidiomycota (100) | Agaricomycetes (100) | Auriculariales (100) | Exidiaceae (100) | Exidia (100) | 4 | 1.72 |
| Fungi (100) | Basidiomycota (100) | Agaricomycetes (100) | Russulales (100) | Peniophoraceae (100) | Peniophoraceae_unclassified (100) | 26 | 11.21 |
| Fungi (100) | Basidiomycota (100) | Agaricomycetes (100) | Polyporales (100) | Schizoporaceae (100) | Xylodon (100) | 14 | 6.03 |
| Fungi (100) | Basidiomycota (100) | Agaricomycetes (100) | Agaricales (100) | Psathyrellaceae (100) | Coprinopsis (100) | 12 | 5.17 |

| **Table S5. Taxonomical classification and abundance of bacterial microbiota members based on 16S rDNA V4 sequences obtained by NGS analysis of total DNA extracted from *C. limon* shoots regenerated under aseptic conditions from surface-sterilized seeds.** Confidence values are enclosed between parentheses. | | | | | | | |
| --- | --- | --- | --- | --- | --- | --- | --- |
| **KINGDOM** | **PHYLUM** | **CLASS** | **ORDER** | **FAMILY** | **GENUS** | **NUMBER OF READS** | **PERCENTAGE** |
| Bacteria(100) | Actinobacteria(100) | Actinobacteria(100) | Propionibacteriales(100) | Propionibacteriaceae(100) | Cutibacterium(100) | 875 | 22.396 |
| Bacteria(100) | Proteobacteria(100) | Gammaproteobacteria(100) | Pseudomonadales(100) | Moraxellaceae(100) | Acinetobacter(100) | 371 | 9.496 |
| Bacteria(100) | Bacteroidetes(100) | Bacteroidia(100) | Cytophagales(100) | Spirosomaceae(100) | Emticicia(100) | 187 | 4.786 |
| Bacteria(100) | Proteobacteria(100) | Gammaproteobacteria(100) | Betaproteobacteriales(100) | Burkholderiaceae(100) | Burkholderiaceae_unclassified(84) | 177 | 4.530 |
| Bacteria(100) | Proteobacteria(100) | Alphaproteobacteria(100) | Sphingomonadales(100) | Sphingomonadaceae(100) | Porphyrobacter(100) | 167 | 4.274 |
| Bacteria(100) | Proteobacteria(100) | Gammaproteobacteria(100) | Xanthomonadales(100) | Xanthomonadaceae(100) | Arenimonas(94) | 139 | 3.558 |
| Bacteria(100) | Proteobacteria(100) | Gammaproteobacteria(100) | Pseudomonadales(100) | Pseudomonadaceae(100) | Pseudomonas(97) | 123 | 3.148 |
| Bacteria(100) | Proteobacteria(100) | Gammaproteobacteria(100) | Alteromonadales(100) | Alteromonadaceae(100) | Rheinheimera(100) | 111 | 2.841 |
| Bacteria(100) | Proteobacteria(100) | Alphaproteobacteria(100) | Sphingomonadales(100) | Sphingomonadaceae(100) | Sphingomonadaceae_unclassified(100) | 84 | 2.150 |
| Bacteria(100) | Proteobacteria(100) | Alphaproteobacteria(100) | Rhizobiales(100) | Rhizobiaceae(100) | Rhizobiaceae_unclassified(100) | 82 | 2.099 |
| Bacteria(100) | Acidobacteria(100) | Subgroup_6(100) | Subgroup_6_or(100) | Subgroup_6_fa(100) | Subgroup_6_ge(100) | 77 | 1.971 |
| Bacteria(100) | Proteobacteria(100) | Gammaproteobacteria(100) | Betaproteobacteriales(100) | Burkholderiaceae(100) | Burkholderiaceae_unclassified(100) | 76 | 1.945 |
| Archaea(100) | Thaumarchaeota(100) | Nitrososphaeria(100) | Nitrososphaerales(100) | Nitrososphaeraceae(100) | Nitrososphaeraceae_unclassified(100) | 72 | 1.843 |
| Bacteria(100) | Proteobacteria(100) | Alphaproteobacteria(100) | Rhizobiales(100) | Hyphomicrobiaceae(100) | Hyphomicrobium(98) | 68 | 1.740 |
| Bacteria(100) | Proteobacteria(100) | Gammaproteobacteria(100) | Betaproteobacteriales(100) | Burkholderiaceae(100) | Herbaspirillum(97) | 64 | 1.638 |
| Bacteria(100) | Bacteroidetes(100) | Bacteroidia(100) | Flavobacteriales(100) | Flavobacteriaceae(100) | Flavobacterium(100) | 59 | 1.510 |
| Bacteria(100) | Chloroflexi(100) | Anaerolineae(100) | Caldilineales(100) | Caldilineaceae(100) | Litorilinea(100) | 49 | 1.254 |
| Bacteria(100) | Firmicutes(100) | Bacilli(100) | Bacillales(100) | Family_XII(100) | Exiguobacterium(100) | 44 | 1.126 |
| Bacteria(100) | Proteobacteria(100) | Gammaproteobacteria(100) | Betaproteobacteriales(100) | Burkholderiaceae(100) | Burkholderiaceae_unclassified(100) | 44 | 1.126 |
| Bacteria(100) | Proteobacteria(100) | Alphaproteobacteria(100) | Rhizobiales(100) | Rhizobiaceae(100) | Rhizobiaceae_unclassified(100) | 41 | 1.049 |
| Bacteria(100) | Actinobacteria(100) | Actinobacteria(100) | Bifidobacteriales(100) | Bifidobacteriaceae(100) | Bifidobacterium(100) | 36 | 0.921 |
| Bacteria(100) | Firmicutes(100) | Bacilli(100) | Lactobacillales(100) | Lactobacillales_unclassified(95) | Lactobacillales_unclassified(95) | 35 | 0.896 |
| Bacteria(100) | Firmicutes(100) | Bacilli(100) | Bacillales(100) | Staphylococcaceae(100) | Staphylococcus(100) | 34 | 0.870 |
| Bacteria(100) | Firmicutes(100) | Bacilli(100) | Bacillales(100) | Bacillaceae(100) | Anaerobacillus(98) | 34 | 0.870 |
| Bacteria(100) | Firmicutes(100) | Clostridia(100) | Clostridiales(100) | Family_XI(100) | Peptoniphilus(100) | 33 | 0.845 |
| Bacteria(100) | Gemmatimonadetes(100) | Gemmatimonadetes(100) | Gemmatimonadales(100) | Gemmatimonadaceae(100) | Gemmatimonadaceae_unclassified(100) | 33 | 0.845 |
| Bacteria(100) | Proteobacteria(100) | Alphaproteobacteria(100) | Sphingomonadales(100) | Sphingomonadaceae(100) | Sphingomonadaceae_unclassified(85) | 32 | 0.819 |
| Bacteria(100) | Gemmatimonadetes(100) | Gemmatimonadetes(100) | Gemmatimonadales(100) | Gemmatimonadaceae(100) | uncultured(100) | 31 | 0.793 |
| Bacteria(100) | Bacteroidetes(100) | Bacteroidia(100) | Chitinophagales(100) | Chitinophagaceae(100) | Flavihumibacter(100) | 27 | 0.691 |
| Bacteria(100) | Proteobacteria(100) | Alphaproteobacteria(100) | Rhizobiales(100) | Beijerinckiaceae(100) | Methylobacterium(100) | 27 | 0.691 |
| Bacteria(100) | Actinobacteria(100) | Actinobacteria(100) | Corynebacteriales(100) | Mycobacteriaceae(100) | Mycobacterium(100) | 26 | 0.665 |
| Bacteria(100) | Proteobacteria(100) | Alphaproteobacteria(100) | Sphingomonadales(100) | Sphingomonadaceae(100) | Sphingomonadaceae_unclassified(100) | 25 | 0.640 |
| Bacteria(100) | Proteobacteria(100) | Gammaproteobacteria(100) | Pseudomonadales(100) | Moraxellaceae(100) | Acinetobacter(100) | 25 | 0.640 |
| Bacteria(100) | Proteobacteria(100) | Gammaproteobacteria(100) | Xanthomonadales(100) | Xanthomonadaceae(100) | Xanthomonas(100) | 25 | 0.640 |
| Bacteria(100) | Bacteria_unclassified(100) | Bacteria_unclassified(100) | Bacteria_unclassified(100) | Bacteria_unclassified(100) | Bacteria_unclassified(100) | 24 | 0.614 |
| Bacteria(100) | Bacteroidetes(100) | Bacteroidia(100) | Cytophagales(100) | Cytophagaceae(100) | Cytophaga(100) | 24 | 0.614 |
| Bacteria(100) | Firmicutes(100) | Bacilli(100) | Bacillales(100) | Bacillales_unclassified(100) | Bacillales_unclassified(100) | 22 | 0.563 |
| Bacteria(100) | Proteobacteria(100) | Gammaproteobacteria(100) | Vibrionales(100) | Vibrionaceae(100) | Vibrionaceae_unclassified(100) | 21 | 0.537 |
| Bacteria(100) | Actinobacteria(100) | Acidimicrobiia(100) | Microtrichales(100) | uncultured(100) | uncultured_ge(100) | 20 | 0.512 |
| Bacteria(100) | Firmicutes(100) | Clostridia(100) | Clostridiales(100) | Ruminococcaceae(100) | Faecalibacterium(100) | 20 | 0.512 |
| Bacteria(100) | Firmicutes(100) | Clostridia(100) | Clostridiales(100) | Family_XI(100) | Anaerococcus(100) | 19 | 0.486 |
| Bacteria(100) | Proteobacteria(100) | Alphaproteobacteria(100) | Rhodobacterales(100) | Rhodobacteraceae(100) | Rhodobacteraceae_unclassified(95) | 19 | 0.486 |
| Bacteria(100) | Actinobacteria(100) | Actinobacteria(100) | Corynebacteriales(100) | Corynebacteriaceae(100) | Corynebacterium_1(95) | 18 | 0.461 |
| Bacteria(100) | Proteobacteria(100) | Gammaproteobacteria(100) | Pseudomonadales(100) | Moraxellaceae(100) | Enhydrobacter(100) | 18 | 0.461 |
| Bacteria(100) | Actinobacteria(100) | Thermoleophilia(100) | Gaiellales(100) | Gaiellales_unclassified(88) | Gaiellales_unclassified(88) | 16 | 0.410 |
| Bacteria(100) | Firmicutes(100) | Bacilli(100) | Lactobacillales(100) | Streptococcaceae(100) | Streptococcus(100) | 16 | 0.410 |
| Bacteria(100) | Actinobacteria(100) | Actinobacteria(100) | Propionibacteriales(100) | Propionibacteriaceae(100) | Cutibacterium(100) | 15 | 0.384 |
| Bacteria(100) | Firmicutes(100) | Bacilli(100) | Lactobacillales(100) | Streptococcaceae(61) | Streptococcaceae_unclassified(61) | 15 | 0.384 |
| Bacteria(100) | Firmicutes(100) | Bacilli(100) | Lactobacillales(100) | Lactobacillaceae(100) | Lactobacillus(100) | 15 | 0.384 |
| Bacteria(100) | Proteobacteria(100) | Alphaproteobacteria(100) | Rhizobiales(100) | Devosiaceae(100) | Devosiaceae_unclassified(100) | 15 | 0.384 |
| Bacteria(100) | Proteobacteria(100) | Alphaproteobacteria(100) | Rhizobiales(100) | Xanthobacteraceae(100) | Xanthobacteraceae_unclassified(94) | 15 | 0.384 |
| Archaea(100) | Thaumarchaeota(100) | Nitrososphaeria(100) | Nitrososphaerales(100) | Nitrososphaeraceae(100) | Nitrososphaeraceae_ge(100) | 15 | 0.384 |
| Bacteria(100) | Acidobacteria(100) | Subgroup_6(100) | Subgroup_6_or(100) | Subgroup_6_fa(100) | Subgroup_6_ge(100) | 14 | 0.358 |
| Bacteria(100) | Proteobacteria(100) | Gammaproteobacteria(100) | Enterobacteriales(100) | Enterobacteriaceae(100) | Enterobacteriaceae_unclassified(75) | 12 | 0.307 |
| Bacteria(100) | Proteobacteria(100) | Gammaproteobacteria(100) | Xanthomonadales(100) | Xanthomonadaceae(100) | Stenotrophomonas(100) | 11 | 0.282 |
| Bacteria(100) | Proteobacteria(100) | Alphaproteobacteria(100) | Rhodobacterales(100) | Rhodobacteraceae(100) | Rhodobacteraceae_unclassified(100) | 10 | 0.256 |
| Bacteria(100) | Firmicutes(100) | Bacilli(100) | Lactobacillales(100) | Streptococcaceae(100) | Streptococcus(100) | 9 | 0.230 |
| Bacteria(100) | Proteobacteria(100) | Alphaproteobacteria(100) | Caulobacterales(100) | Caulobacteraceae(100) | Brevundimonas(100) | 9 | 0.230 |
| Bacteria(100) | Firmicutes(100) | Bacilli(100) | Bacillales(100) | Bacillaceae(100) | Anaerobacillus(88) | 8 | 0.205 |
| Bacteria(100) | Proteobacteria(100) | Gammaproteobacteria(100) | Pasteurellales(100) | Pasteurellaceae(100) | Haemophilus(75) | 8 | 0.205 |
| Bacteria(100) | Proteobacteria(100) | Alphaproteobacteria(100) | Acetobacterales(100) | Acetobacteraceae(100) | Roseomonas(100) | 8 | 0.205 |
| Bacteria(100) | Actinobacteria(100) | Actinobacteria(100) | Micrococcales(100) | Micrococcaceae(100) | Glutamicibacter(100) | 7 | 0.179 |
| Bacteria(100) | Actinobacteria(100) | Thermoleophilia(100) | Gaiellales(100) | Gaiellales_unclassified(100) | Gaiellales_unclassified(100) | 6 | 0.154 |
| Bacteria(100) | Actinobacteria(100) | Actinobacteria(100) | Propionibacteriales(100) | Nocardioidaceae(100) | Nocardioides(100) | 5 | 0.128 |
| Bacteria(100) | Actinobacteria(100) | Actinobacteria(100) | Micrococcales(100) | Brevibacteriaceae(100) | Brevibacterium(100) | 5 | 0.128 |
| Bacteria(100) | Firmicutes(100) | Bacilli(100) | Bacillales(100) | Alicyclobacillaceae(100) | Tumebacillus(100) | 5 | 0.128 |
| Bacteria(100) | Gemmatimonadetes(100) | Gemmatimonadetes(100) | Gemmatimonadales(100) | Gemmatimonadaceae(100) | Gemmatimonas(100) | 5 | 0.128 |
| Bacteria(100) | Planctomycetes(100) | Phycisphaerae(100) | Tepidisphaerales(100) | WD2101_soil_group(100) | WD2101_soil_group_ge(100) | 5 | 0.128 |
| Bacteria(100) | Verrucomicrobia(100) | Verrucomicrobiae(100) | Chthoniobacterales(100) | Chthoniobacteraceae(100) | Chthoniobacter(100) | 5 | 0.128 |
| Bacteria(100) | Actinobacteria(100) | Actinobacteria(100) | Corynebacteriales(100) | Nocardiaceae(100) | Rhodococcus(100) | 4 | 0.102 |
| Bacteria(100) | Actinobacteria(100) | Actinobacteria(100) | Corynebacteriales(100) | Corynebacteriaceae(100) | Corynebacterium_1(100) | 4 | 0.102 |
| Bacteria(100) | Proteobacteria(100) | Gammaproteobacteria(100) | Alteromonadales(100) | Shewanellaceae(100) | Shewanella(100) | 4 | 0.102 |
| Bacteria(100) | Acidobacteria(100) | Subgroup_6(100) | Subgroup_6_or(100) | Subgroup_6_fa(100) | Subgroup_6_ge(100) | 3 | 0.077 |
| Bacteria(100) | Actinobacteria(100) | Actinobacteria(100) | Corynebacteriales(100) | Corynebacteriaceae(100) | Lawsonella(100) | 3 | 0.077 |
| Bacteria(100) | Bacteroidetes(100) | Bacteroidia(100) | Flavobacteriales(100) | Weeksellaceae(100) | Empedobacter(100) | 3 | 0.077 |
| Bacteria(100) | Bacteroidetes(100) | Bacteroidia(100) | Flavobacteriales(100) | NS9_marine_group(100) | NS9_marine_group_ge(100) | 3 | 0.077 |
| Bacteria(100) | Chloroflexi(100) | Anaerolineae(100) | Caldilineales(100) | Caldilineaceae(100) | Litorilinea(100) | 3 | 0.077 |
| Bacteria(100) | Firmicutes(100) | Bacilli(100) | Bacillales(100) | Planococcaceae(100) | Planococcaceae_unclassified(100) | 3 | 0.077 |
| Bacteria(100) | Proteobacteria(100) | Gammaproteobacteria(100) | Pseudomonadales(100) | Moraxellaceae(100) | Psychrobacter(100) | 3 | 0.077 |
| Bacteria(100) | Proteobacteria(100) | Gammaproteobacteria(100) | Oceanospirillales(100) | Saccharospirillaceae(100) | Bermanella(67) | 3 | 0.077 |
| Bacteria(100) | Proteobacteria(100) | Alphaproteobacteria(100) | Sphingomonadales(100) | Sphingomonadaceae(100) | Rhizorhapis(100) | 3 | 0.077 |
| Bacteria(100) | Proteobacteria(100) | Gammaproteobacteria(100) | Betaproteobacteriales(100) | Burkholderiaceae(100) | Burkholderia-Caballeronia-Paraburkholderia(100) | 3 | 0.077 |
| Bacteria(100) | Proteobacteria(100) | Gammaproteobacteria(100) | Alteromonadales(100) | Shewanellaceae(100) | Shewanellaceae_unclassified(67) | 3 | 0.077 |
| Bacteria(100) | Acidobacteria(100) | Subgroup_6(100) | Subgroup_6_or(100) | Subgroup_6_fa(100) | Subgroup_6_ge(100) | 2 | 0.051 |
| Bacteria(100) | Actinobacteria(100) | Actinobacteria(100) | Streptomycetales(100) | Streptomycetaceae(100) | Streptomyces(100) | 2 | 0.051 |
| Bacteria(100) | Actinobacteria(100) | Actinobacteria(100) | Micrococcales(100) | Micrococcaceae(100) | Micrococcaceae_unclassified(100) | 2 | 0.051 |
| Bacteria(100) | Actinobacteria(100) | Acidimicrobiia(100) | Microtrichales(100) | uncultured(100) | uncultured_ge(100) | 2 | 0.051 |
| Bacteria(100) | Actinobacteria(100) | Acidimicrobiia(100) | Microtrichales(100) | uncultured(100) | uncultured_ge(100) | 2 | 0.051 |
| Bacteria(100) | Actinobacteria(100) | Rubrobacteria(100) | Rubrobacterales(100) | Rubrobacteriaceae(100) | Rubrobacter(100) | 2 | 0.051 |
| Bacteria(100) | Actinobacteria(100) | Actinobacteria(100) | Micrococcales(100) | Microbacteriaceae(100) | Microbacterium(100) | 2 | 0.051 |
| Bacteria(100) | Firmicutes(100) | Bacilli(100) | Bacillales(100) | Paenibacillaceae(100) | Saccharibacillus(100) | 2 | 0.051 |
| Bacteria(100) | Fusobacteria(100) | Fusobacteriia(100) | Fusobacteriales(100) | Fusobacteriaceae(100) | Propionigenium(100) | 2 | 0.051 |
| Bacteria(100) | Proteobacteria(100) | Gammaproteobacteria(100) | Pseudomonadales(100) | Moraxellaceae(100) | Acinetobacter(100) | 2 | 0.051 |
| Bacteria(100) | Proteobacteria(100) | Gammaproteobacteria(100) | Gammaproteobacteria_unclassified(100) | Gammaproteobacteria_unclassified(100) | Gammaproteobacteria_unclassified(100) | 2 | 0.051 |
| Bacteria(100) | Proteobacteria(100) | Gammaproteobacteria(100) | Betaproteobacteriales(100) | Burkholderiaceae(100) | Burkholderiaceae_unclassified(100) | 2 | 0.051 |
| Bacteria(100) | Proteobacteria(100) | Gammaproteobacteria(100) | Vibrionales(100) | Vibrionaceae(100) | Vibrionaceae_unclassified(100) | 2 | 0.051 |
| Bacteria(100) | Proteobacteria(100) | Gammaproteobacteria(100) | Cellvibrionales(100) | Cellvibrionaceae(100) | Cellvibrio(100) | 2 | 0.051 |
| Bacteria(100) | Proteobacteria(100) | Alphaproteobacteria(100) | Rhizobiales(100) | Xanthobacteraceae(100) | Xanthobacteraceae_unclassified(100) | 2 | 0.051 |
| Bacteria(100) | Proteobacteria(100) | Alphaproteobacteria(100) | Rhodobacterales(100) | Rhodobacteraceae(100) | Pseudophaeobacter(100) | 2 | 0.051 |
| Bacteria(100) | Proteobacteria(100) | Alphaproteobacteria(100) | Caulobacterales(100) | Caulobacteraceae(100) | Caulobacter(100) | 2 | 0.051 |
| Bacteria(100) | Acidobacteria(100) | Subgroup_6(100) | Subgroup_6_or(100) | Subgroup_6_fa(100) | Subgroup_6_ge(100) | 1 | 0.026 |
| Bacteria(100) | Acidobacteria(100) | Subgroup_6(100) | Subgroup_6_or(100) | Subgroup_6_fa(100) | Subgroup_6_ge(100) | 1 | 0.026 |
| Bacteria(100) | Acidobacteria(100) | Subgroup_6(100) | Subgroup_6_or(100) | Subgroup_6_fa(100) | Subgroup_6_ge(100) | 1 | 0.026 |
| Bacteria(100) | Acidobacteria(100) | Subgroup_6(100) | Subgroup_6_or(100) | Subgroup_6_fa(100) | Subgroup_6_ge(100) | 1 | 0.026 |
| Bacteria(100) | Actinobacteria(100) | Actinobacteria(100) | Micrococcales(100) | Micrococcaceae(100) | Kocuria(100) | 1 | 0.026 |
| Bacteria(100) | Actinobacteria(100) | Actinobacteria(100) | Corynebacteriales(100) | Nocardiaceae(100) | Gordonia(100) | 1 | 0.026 |
| Bacteria(100) | Chloroflexi(100) | Anaerolineae(100) | Caldilineales(100) | Caldilineaceae(100) | Litorilinea(100) | 1 | 0.026 |
| Bacteria(100) | Chloroflexi(100) | Anaerolineae(100) | Caldilineales(100) | Caldilineaceae(100) | Litorilinea(100) | 1 | 0.026 |
| Bacteria(100) | Chloroflexi(100) | Anaerolineae(100) | Caldilineales(100) | Caldilineaceae(100) | Litorilinea(100) | 1 | 0.026 |
| Bacteria(100) | Chloroflexi(100) | Anaerolineae(100) | Caldilineales(100) | Caldilineaceae(100) | Litorilinea(100) | 1 | 0.026 |
| Bacteria(100) | Chloroflexi(100) | Anaerolineae(100) | Caldilineales(100) | Caldilineaceae(100) | Litorilinea(100) | 1 | 0.026 |
| Bacteria(100) | Chloroflexi(100) | Anaerolineae(100) | Caldilineales(100) | Caldilineaceae(100) | Litorilinea(100) | 1 | 0.026 |
| Bacteria(100) | Chloroflexi(100) | Anaerolineae(100) | Caldilineales(100) | Caldilineaceae(100) | Litorilinea(100) | 1 | 0.026 |
| Bacteria(100) | Firmicutes(100) | Clostridia(100) | Clostridiales(100) | Lachnospiraceae(100) | Blautia(100) | 1 | 0.026 |
| Bacteria(100) | Firmicutes(100) | Clostridia(100) | Clostridiales(100) | Ruminococcaceae(100) | Ruminococcaceae_UCG-005(100) | 1 | 0.026 |
| Bacteria(100) | Fusobacteria(100) | Fusobacteriia(100) | Fusobacteriales(100) | Fusobacteriaceae(100) | Propionigenium(100) | 1 | 0.026 |
| Bacteria(100) | Fusobacteria(100) | Fusobacteriia(100) | Fusobacteriales(100) | Fusobacteriaceae(100) | Propionigenium(100) | 1 | 0.026 |
| Bacteria(100) | Fusobacteria(100) | Fusobacteriia(100) | Fusobacteriales(100) | Fusobacteriaceae(100) | Propionigenium(100) | 1 | 0.026 |
| Bacteria(100) | Planctomycetes(100) | Phycisphaerae(100) | Tepidisphaerales(100) | WD2101_soil_group(100) | WD2101_soil_group_ge(100) | 1 | 0.026 |
| Bacteria(100) | Proteobacteria(100) | Gammaproteobacteria(100) | Aeromonadales(100) | Aeromonadaceae(100) | Aeromonas(100) | 1 | 0.026 |
| Bacteria(100) | Proteobacteria(100) | Gammaproteobacteria(100) | Alteromonadales(100) | Pseudoalteromonadaceae(100) | Pseudoalteromonas(100) | 1 | 0.026 |
| Bacteria(100) | Proteobacteria(100) | Alphaproteobacteria(100) | Rhodobacterales(100) | Rhodobacteraceae(100) | Rhodobacteraceae_unclassified(100) | 1 | 0.026 |
| Bacteria(100) | Proteobacteria(100) | Gammaproteobacteria(100) | Vibrionales(100) | Vibrionaceae(100) | Photobacterium(100) | 1 | 0.026 |
| Bacteria(100) | Proteobacteria(100) | Gammaproteobacteria(100) | Betaproteobacteriales(100) | Burkholderiaceae(100) | Burkholderiaceae_unclassified(100) | 1 | 0.026 |
| Bacteria(100) | Proteobacteria(100) | Gammaproteobacteria(100) | Alteromonadales(100) | Alteromonadaceae(100) | Rheinheimera(100) | 1 | 0.026 |
| Bacteria(100) | Proteobacteria(100) | Gammaproteobacteria(100) | Betaproteobacteriales(100) | Burkholderiaceae(100) | Massilia(100) | 1 | 0.026 |
| Bacteria(100) | Proteobacteria(100) | Gammaproteobacteria(100) | Pseudomonadales(100) | Pseudomonadaceae(100) | Pseudomonadaceae_unclassified(100) | 1 | 0.026 |
| Bacteria(100) | Proteobacteria(100) | Gammaproteobacteria(100) | Betaproteobacteriales(100) | Burkholderiaceae(100) | Burkholderiaceae_unclassified(100) | 1 | 0.026 |
| Bacteria(100) | Proteobacteria(100) | Gammaproteobacteria(100) | Betaproteobacteriales(100) | Chromobacteriaceae(100) | Vogesella(100) | 1 | 0.026 |
| Bacteria(100) | Proteobacteria(100) | Gammaproteobacteria(100) | Oceanospirillales(100) | Alcanivoracaceae(100) | Alcanivorax(100) | 1 | 0.026 |
| Bacteria(100) | Proteobacteria(100) | Alphaproteobacteria(100) | Rhodobacterales(100) | Rhodobacteraceae(100) | Rhodobacteraceae_unclassified(100) | 1 | 0.026 |
| Bacteria(100) | Proteobacteria(100) | Alphaproteobacteria(100) | Rhodobacterales(100) | Rhodobacteraceae(100) | Rhodobacteraceae_unclassified(100) | 1 | 0.026 |
| Bacteria(100) | Proteobacteria(100) | Alphaproteobacteria(100) | Rhizobiales(100) | Hyphomicrobiaceae(100) | Hyphomicrobiaceae_unclassified(100) | 1 | 0.026 |
| Archaea(100) | Thaumarchaeota(100) | Nitrososphaeria(100) | Nitrososphaerales(100) | Nitrososphaeraceae(100) | Nitrososphaeraceae_ge(100) | 1 | 0.026 |
| Archaea(100) | Thaumarchaeota(100) | Nitrososphaeria(100) | Nitrososphaerales(100) | Nitrososphaeraceae(100) | Nitrososphaeraceae_ge(100) | 1 | 0.026 |
| Bacteria(100) | Verrucomicrobia(100) | Verrucomicrobiae(100) | Chthoniobacterales(100) | Chthoniobacteraceae(100) | Chthoniobacter(100) | 1 | 0.026 |

| **Table S6. Taxonomical classification and abundance of fungal microbiota members based on ITS2 sequences obtained by NGS analysis of total DNA extracted from shoots regenerated under aseptic conditions from surface-sterilized seeds.** Confidence values are enclosed between parentheses. | | | | | | | |
| --- | --- | --- | --- | --- | --- | --- | --- |
| **KINGDOM** | **PHYLUM** | **CLASS** | **ORDER** | **FAMILY** | **GENUS** | **NUMBER OF READS** | **PERCENTAGE** |
| Fungi (100) | Ascomycota (100) | Dothideomycetes (100) | Capnodiales (100) | Capnodiales_unclassified (97) | Capnodiales_unclassified (97) | 994 | 35.82 |
| Fungi (100) | Ascomycota (100) | Saccharomycetes (100) | Saccharomycetales (100) | Debaryomycetaceae (100) | Debaryomyces (100) | 482 | 17.37 |
| Fungi (100) | Ascomycota (100) | Eurotiomycetes (100) | Eurotiales (100) | Aspergillaceae (100) | Penicillium (100) | 297 | 10.70 |
| Fungi (100) | Ascomycota (100) | Saccharomycetes (100) | Saccharomycetales (100) | Saccharomycetales_fam_Incertae_sedis (100) | Candida (100) | 151 | 5.44 |
| Fungi (100) | Basidiomycota (100) | Tremellomycetes (100) | Filobasidiales (100) | Filobasidiaceae (100) | Filobasidium (100) | 135 | 4.86 |
| Fungi (100) | Ascomycota (100) | Dothideomycetes (100) | Pleosporales (100) | Lophiostomataceae (100) | unclassified_Lophiostomataceae (100) | 130 | 4.68 |
| Fungi (100) | Fungi_unclassified (100) | Fungi_unclassified (100) | Fungi_unclassified (100) | Fungi_unclassified (100) | Fungi_unclassified (100) | 85 | 3.06 |
| Fungi (100) | Ascomycota (100) | Sordariomycetes (100) | Hypocreales (100) | Nectriaceae (100) | Nectriaceae_unclassified (100) | 66 | 2.38 |
| Fungi (100) | Ascomycota (100) | Dothideomycetes (100) | Pleosporales (100) | Pleosporales_unclassified (100) | Pleosporales_unclassified (100) | 64 | 2.31 |
| Fungi (100) | Basidiomycota (100) | Agaricomycetes (100) | Thelephorales (100) | Thelephoraceae (100) | Pseudotomentella (100) | 58 | 2.09 |
| Fungi (100) | Ascomycota (100) | Sordariomycetes (100) | Glomerellales (100) | Plectosphaerellaceae (100) | Plectosphaerella (100) | 56 | 2.02 |
| Fungi (100) | Ascomycota (100) | Dothideomycetes (100) | Pleosporales (100) | Pleosporaceae (100) | Alternaria (100) | 40 | 1.44 |
| Fungi (100) | Ascomycota (100) | Pezizomycetes (100) | Pezizales (100) | Pyronemataceae (100) | Picoa (100) | 38 | 1.37 |
| Fungi (100) | Basidiomycota (100) | Malasseziomycetes (100) | Malasseziales (100) | Malasseziaceae (100) | Malassezia (100) | 30 | 1.08 |
| Fungi (100) | Basidiomycota (100) | Tremellomycetes (100) | Tremellales (100) | Bulleribasidiaceae (100) | Vishniacozyma (100) | 23 | 0.83 |
| Fungi (100) | Ascomycota (100) | Sordariomycetes (100) | Hypocreales (100) | Stachybotryaceae (100) | Stachybotrys (100) | 22 | 0.79 |
| Fungi (100) | Ascomycota (100) | Eurotiomycetes (100) | Chaetothyriales (100) | Herpotrichiellaceae (100) | Exophiala (100) | 20 | 0.72 |
| Fungi (100) | Basidiomycota (100) | Malasseziomycetes (100) | Malasseziales (100) | Malasseziaceae (100) | Malassezia (100) | 16 | 0.58 |
| Fungi (100) | Fungi_unclassified (100) | Fungi_unclassified (100) | Fungi_unclassified (100) | Fungi_unclassified (100) | Fungi_unclassified (100) | 9 | 0.32 |
| Fungi (100) | Ascomycota (100) | Eurotiomycetes (100) | Eurotiales (100) | Aspergillaceae (100) | Penicillium (100) | 8 | 0.29 |
| Fungi (100) | Fungi_unclassified (100) | Fungi_unclassified (100) | Fungi_unclassified (100) | Fungi_unclassified (100) | Fungi_unclassified (100) | 8 | 0.29 |
| Fungi (100) | Basidiomycota (100) | Agaricomycetes (100) | Polyporales (100) | Schizophyllaceae (100) | Schizophyllum (100) | 7 | 0.25 |
| Fungi (100) | Ascomycota (100) | Sordariomycetes (100) | Hypocreales (100) | Nectriaceae (100) | Fusarium (100) | 7 | 0.25 |
| Fungi (100) | Basidiomycota (100) | Malasseziomycetes (100) | Malasseziales (100) | Malasseziaceae (100) | Malassezia (100) | 3 | 0.11 |
| Fungi (100) | Basidiomycota (100) | Malasseziomycetes (100) | Malasseziales (100) | Malasseziaceae (100) | Malassezia (100) | 3 | 0.11 |
| Fungi (100) | Ascomycota (100) | Pezizomycetes (100) | Pezizales (100) | Ascobolaceae (100) | unclassified_Ascobolaceae (100) | 3 | 0.11 |
| Fungi (100) | Fungi_unclassified (100) | Fungi_unclassified (100) | Fungi_unclassified (100) | Fungi_unclassified (100) | Fungi_unclassified (100) | 3 | 0.11 |
| Fungi (100) | Fungi_unclassified (100) | Fungi_unclassified (100) | Fungi_unclassified (100) | Fungi_unclassified (100) | Fungi_unclassified (100) | 3 | 0.11 |
| Fungi (100) | Basidiomycota (100) | Malasseziomycetes (100) | Malasseziales (100) | Malasseziaceae (100) | Malassezia (100) | 2 | 0.07 |
| Fungi (100) | Ascomycota (100) | Dothideomycetes (100) | Capnodiales (100) | Capnodiales_unclassified (100) | Capnodiales_unclassified (100) | 2 | 0.07 |
| Fungi (100) | Fungi_unclassified (100) | Fungi_unclassified (100) | Fungi_unclassified (100) | Fungi_unclassified (100) | Fungi_unclassified (100) | 2 | 0.07 |
| Fungi (100) | Fungi_unclassified (100) | Fungi_unclassified (100) | Fungi_unclassified (100) | Fungi_unclassified (100) | Fungi_unclassified (100) | 2 | 0.07 |
| Fungi (100) | Fungi_unclassified (100) | Fungi_unclassified (100) | Fungi_unclassified (100) | Fungi_unclassified (100) | Fungi_unclassified (100) | 2 | 0.07 |
| Fungi (100) | Fungi_unclassified (100) | Fungi_unclassified (100) | Fungi_unclassified (100) | Fungi_unclassified (100) | Fungi_unclassified (100) | 2 | 0.07 |
| Fungi (100) | Fungi_unclassified (100) | Fungi_unclassified (100) | Fungi_unclassified (100) | Fungi_unclassified (100) | Fungi_unclassified (100) | 1 | 0.04 |
| Fungi (100) | Fungi_unclassified (100) | Fungi_unclassified (100) | Fungi_unclassified (100) | Fungi_unclassified (100) | Fungi_unclassified (100) | 1 | 0.04 |

| **Table S7. Alpha diversity indices** | | | | | | | | | |
| --- | --- | --- | --- | --- | --- | --- | --- | --- | --- |
| **Label** | **Group** | **ace** | **chao** | **sobs** | **Simpson** | **invsimpson** | **shannon** | **nseqs** | **coverage** |
| 16S | SHOOT | 179.96 | 171 | 136 | 0.073 | 13.73 | 3.51 | 3907 | 0.991 |
| 16S | SEED | 122.77 | 134.1 | 99 | 0.103 | 9.70 | 3.01 | 2386 | 0.989 |
| ITS2 | SHOOT | 37.08 | 36.1 | 36 | 0.181 | 5.53 | 2.27 | 2775 | 0.999 |
| ITS2 | SEED | 21.32 | 23 | 20 | 0.089 | 11.28 | 2.65 | 232 | 0.987 |

| **Table S8. Beta diversity indices** | | | |
| --- | --- | --- | --- |
| **Sample** | **jclass** | **thetayc** |  |
| Bacteria | 0.820 | 0.718 |  |
| Fungi | 0.857 | 0.771 |  |

**Table S9 PERMANOVA statistical test performed between seeds and shoots for bacterial or fungal microbiota structures.** JCLASS and thetaYC distances were used with 999 permutations.

|  | **pseudo-F (*p*)** | |
| --- | --- | --- |
| **Kingdom** | **JCLASS** | **thetaYC** |
| Fungi | 1.36 (0.099) | 0.96 (0.482) |
| Bacteria | 1.22 (0.094) | 1.25 (0.379) |
